# High density-lipoprotein regulates liquid-liquid phase separation of heat shock protein β-1 by lncRNA HDRACA to affect vascular inflammation and atherosclerosis

**DOI:** 10.7150/ijbs.135145

**Published:** 2026-07-01

**Authors:** Zhi-Wei Mo, Yi-Fang Liu, Yi-Xin Zhang, Yan Li, Yang Cao, Chu-Yu Liu, Le Li, Yue-Ting Kang, Hong-Yu Cao, Zhen-Sheng Ma, Yue-Ming Peng, Zhi-Jun Ou, Guang-Qi Chang, Jing-Song Ou

**Affiliations:** 1 Division of Cardiac Surgery, Cardiovascular Diseases Institute, The First Affiliated Hospital, Sun Yat-sen University, Guangzhou, 510080, P.R. China.; 2 Division of Vascular Surgery, The First Affiliated Hospital, Sun Yat-sen University, Guangzhou, 510080, P.R. China.; 3 National-Guangdong Joint Engineering Laboratory for Diagnosis and Treatment of Vascular Diseases, NHC key Laboratory of Assisted Circulation and Vascular Diseases (Sun Yat-sen University), Key Laboratory of Assisted Circulation and Vascular Diseases, Chinese Academy of Medical Sciences, Guangdong Provincial Engineering and Technology Center for Diagnosis and Treatment of Vascular Diseases, Guangzhou, 510080, P.R. China.; 4 Division of Hypertension and Vascular Diseases, Department of Cardiology, Cardiovascular Diseases Institute, The First Affiliated Hospital, Sun Yat-sen University, Guangzhou, 510080, P.R. China.; 5 Guangdong Provincial Key Laboratory of Brain Function and Disease, Zhongshan School of Medicine, Sun Yat-sen University, Guangzhou, 510080, P.R. China.; 6 Department of Biomedical Sciences, Faculty of Health Sciences, University of Macau, Taipa, Macau SAR, P.R. China.

**Keywords:** high-density lipoprotein, inflammation, long noncoding RNA, liquid-liquid phase separation, atherosclerosis

## Abstract

High-density lipoprotein (HDL) from healthy subjects (HDL_healthy_) has anti-inflammatory effects, whereas HDL from patients with coronary artery disease (HDL_CAD_) is functionally impaired, which may affect atherosclerosis formation differently. We previously demonstrated that HDL_healthy_ inhibits the expression of the long noncoding RNA *high-density lipoprotein-regulated angiogenesis in coronary artery disease* (HDRACA), whereas HDL_CAD_ is much less effective. HDRACA is highly expressed in endothelial cells (ECs) of arteriosclerosis obliterans and is associated with the expression of adhesion molecules and chemokines. However, whether HDL_healthy_ and HDL_CAD_ affect vascular inflammation and atherosclerosis by regulating HDRACA expression remains unclear. Here, we found that HDL_healthy_ suppressed HDRACA expression in ECs, thereby inhibiting endothelial chemotactic and adhesive effects, whereas HDL_CAD_ had less of an effect. Mechanistically, HDRACA bound to heat shock protein β-1 (HSPB1), an anti-inflammatory protein that underwent liquid-liquid phase separation (LLPS) mediated by its N-terminal domain (NTD) and C-terminal domain (CTD). Overexpression of HDRACA promoted LLPS of HSPB1, inhibited HSPB1-IKKβ interaction, and activated the NF-ĸB pathway, leading to increased expression of adhesion molecules and chemokines. HDL_healthy_ enhanced the phosphorylation of serine residues 15, 78, and 82 in the NTD of HSPB1 to inhibit its LLPS by suppressing HDRACA expression, whereas HDL_CAD_ was less effective. Delivering HDRACA into mouse aortic ECs also promoted LLPS of HSPB1, leading to enhanced vascular inflammation and accelerated atherosclerosis formation in low-density lipoprotein receptor null mice. Our findings identified HDRACA-induced LLPS of HSPB1 as a novel mechanism linking HDL dysfunction and atherosclerosis development. HDL_healthy_ suppressed HDRACA-induced LLPS of HSPB1 to inhibit vascular inflammation and exerted anti-atherosclerotic effects, whereas HDL_CAD_ lost its protective role because of its inefficacy in HDRACA inhibition. These findings suggest that improving HDL function, inhibiting HDRACA, and blocking LLPS of HSPB1 may represent promising therapeutic strategies for atherosclerosis.

## Introduction

Atherosclerosis is a core vascular pathological change associated with atherosclerotic cardiovascular disease (ASCVD). Traditional risk factors, including hypercholesterolemia, diabetes mellitus, smoking, and obesity, can cause persistent production of oxidative stress, inflammatory factors, and oxidized lipids, all of which contribute to the development of atherosclerosis 1-4. Recent studies have confirmed that risk factors, including mental stress, dysbiosis, and viral infections, also induce the production of inflammatory factors, thereby increasing the risk of atherosclerosis 5-7. These proinflammatory mediators can cause dysfunction of endothelial cells (ECs) that sustainably express adhesion molecules and chemokines, which induce immune cells to continuously infiltrate the vascular wall, further causing atherosclerosis progression 2, 4, 8. In healthy subjects, high density-lipoprotein (HDL) exerts anti-atherogenic effects by inhibiting the adhesive and chemotactic activities of ECs induced by proinflammatory mediators 9-11. However, we and other researchers found that HDL undergoes multiple modifications, losing its protective ability and becoming proinflammatory or dysfunctional in ASCVD 9. While previous studies have primarily focused on the compositional alterations of HDL in ASCVD, the mechanistic changes underlying HDL-mediated regulation of endothelial adhesion and chemotaxis in ASCVD remain poorly understood.

Long noncoding RNA (lncRNA) is arbitrarily defined as a class of transcripts comprising more than 200 nucleotides with limited protein-coding potential 12. Recent studies have demonstrated that lncRNAs participate in the development of atherosclerosis 13-16. We previously demonstrated that HDL from healthy subjects (HDL_healthy_) inhibits the expression of lncRNA *high-density lipoprotein-regulated angiogenesis in coronary artery disease* (HDRACA) in ECs, whereas HDL from patients with coronary artery disease (HDL_CAD_) is much less effective because of sphingosine 1-phosphate (S1P) deficiency 17. Specifically, HDRACA is upregulated in the arterial ECs of patients with arteriosclerosis obliterans, and its silencing alters the expression of genes associated with the TNF signaling, cell adhesion, and chemokine signaling pathways 17. Additionally, we found putative interactions between HDRACA and multiple proteins, most notably the highly enriched heat shock protein β-1 (HSPB1) 17. HSPB1, a canonical small heat shock protein (sHSP), has been recognized for its anti-inflammatory effects and role in stabilizing atherosclerotic plaques 18-20. A previous study demonstrated that HSPB1 overexpression effectively decreases *de novo* atherosclerotic lesion formation and increases plaque stability 21. HSPB1 can interact with the inhibitor of κB kinase (IKK) complex, thereby inhibiting inhibitor of κB α (IκBα) degradation and nuclear factor-ĸB (NF-κB) nuclear translocation, ultimately suppressing the expression of adhesion molecules and chemotaxis-related genes 22. Thus, the distinct regulatory effects of HDL_healthy_ and HDL_CAD_ on the HDRACA/HSPB1/IKKβ/IκBα/NF-κB pathway may account for their different anti-inflammatory properties.

The concept of liquid-liquid phase separation (LLPS) is relatively novel in the field of biomedicine. It refers to the process that biological polymers including DNA, RNA and proteins form liquid-like condensates through weak multivalent interactions, which dynamically coexists with a dilute phase, enabling spatial organization of biomolecules 23. This either concentrates functionally related biomolecules to activate signal transduction or sequesters them to inhibit interactions 23. Recent studies found that LLPS of proteins mediates vascular inflammation and atherosclerosis formation 24, 25. Increasing evidence highlights that lncRNAs are important drivers of LLPS-mediated regulation of protein activity 26, 27. However, it is unclear whether HDL_healthy_ and HDL_CAD_ differentially affect atherosclerosis by regulating LLPS-mediated protein activity via lncRNAs.

In the present study, we found that HDL_healthy_ downregulated HDRACA to inhibit LLPS of HSPB1 in ECs, whereas HDL_CAD_ was much less effective. Non-aggregated HSPB1 interacted with IKKβ to inhibit NF-κB signaling activation and suppressed the expression of adhesion molecules and chemokines, thus attenuating the adhesive and chemotactic effects of ECs and ultimately inhibiting atherosclerosis formation. Our findings reveal that HDL_healthy_ may inhibit atherosclerosis by attenuating HDRACA-induced LLPS of HSPB1, whereas HDL_CAD_ cannot effectively inhibit atherosclerosis because it is less effective in suppressing HDRACA expression, which provides a novel entry point for developing effective anti-inflammatory-based therapeutics for atherosclerosis.

## Materials and Methods

More detailed materials and methods are available in the Supplementary Materials.

### Study populations and HDL isolation

Age- and sex-matched patients with CAD and healthy subjects without cardiovascular risk factors were enrolled to isolate HDL. To avoid the confounding effects of medications, the enrolled patients were all newly diagnosed with CAD and had not received any drug treatment in the three months prior to HDL isolation. Detailed information on the patients and healthy volunteers is provided in the Supplementary Table S1. The levels of S1P in HDL were quantified using a previously described method 17. Written informed consent was obtained from all the participants. Plasma was isolated from individual venous blood samples and then subjected to HDL isolation through sequential ultracentrifugation, as described previously 17, 28-30. For functional and molecular biological assays involving HDL, HDL preparations were randomly selected from the HDL sample pool. The study protocol was approved by the Ethics Review Board of the First Affiliated Hospital, Sun Yat-sen University (approval number: [2023]374) and was conducted in accordance with the principles outlined in the Declaration of Helsinki.

### Assessment of nHDLox

HDL lipid peroxide content (HDLox) was measured, and normalized HDLox (nHDLox) was calculated to assess HDL function, as described previously 31.

### Cell culture

Human umbilical vein endothelial cells (HUVECs), mouse aortic endothelial cells (MAECs), and THP-1 monocytes were cultured at 37 °C in a humidified incubator with 5% CO_2_. HUVECs were serum-starved overnight prior to treatment with 100 μg protein/mL HDL_healthy_ or HDL_CAD_, followed by incubation with 100 ng/mL tumor necrosis factor-α (TNF-α). The concentration used in this study was based on the HDL concentrations used in our previous studies, which approximate physiological levels in human plasma 17, 29, 32.

### Adhesion assay and chemotaxis assay

Adhesion and chemotaxis assays were performed to evaluate adhesive and chemotactic effects of ECs.

### RNAi and antisense silencing studies

Cells were transfected with lncRNA Smart Silencers or small interfering RNAs (siRNAs) following the manufacturer's instructions for the Lipofectamine RNAiMAX Transfection Reagent. The target sequences of lncRNA Smart Silencers and siRNAs are listed in the Supplementary Table S2.

### Lentiviral transduction

Cells at 30-50% confluence were exposed to lentiviral particles and cultured for 72 h prior to subsequent experiments unless otherwise indicated. Enhanced green fluorescent protein (EGFP) linked HSPB1 wild-type or mutants were expressed in ECs via lentiviral transduction. To reduce potential interference between EGFP and wild-type or mutant HSPB1 domains, a linker was inserted between them to maintain their independent functions 33.

### RNA extraction, reverse transcription, and RT-qPCR

Total RNA was extracted and reverse-transcribed into complementary DNA. Real-time quantitative polymerase chain reaction (RT-qPCR) was performed as described previously 17. Primers used for RT-qPCR are listed in the Supplementary Table S3.

### Western blot

The western blot assays were performed as described previously 34-37.

### Enzyme-linked immunosorbent assay

The levels of monocyte chemoattractant protein-1 (MCP-1) in the culture medium of HUVECs and MAECs were measured using enzyme-linked immunosorbent assays (ELISA).

### Immunoprecipitation

The immunoprecipitation assays were performed as described previously 38, 39.

### Nuclear/cytoplasmic fractionation

Nuclear and cytoplasmic fractions were isolated using NE-PER Nuclear and Cytoplasmic Extraction Reagents following the manufacturer's instructions.

### RNA pull-down assay

RNA pull-down assays were performed according to the manufacturer's instructions for the Pierce™ Magnetic RNA-Protein Pull-Down Kit, as described previously 17.

### RNA immunoprecipitation

RNA immunoprecipitation (RIP) assays were performed according to the manufacturer's instructions for the Magna RIP™ RNA-Binding Protein Immunoprecipitation Kit, as described previously 40, 41.

### Fluorescence *in situ* hybridization and immunofluorescence assays

Fluorescence *in situ* hybridization (FISH) assays were performed to analyze the expression of HDRACA in HUVECs and aortic roots, according to the manufacturer's instructions for the Ribo^TM^ Fluorescent *In Situ* Hybridization Kit. Immunofluorescence staining was performed to detect HSPB1 expression in HUVECs, MAECs, and VE-cadherin-positive cells in whole-mount en face mouse aortic preparations. Additionally, vascular cell adhesion molecule 1 (VCAM1), intercellular adhesion molecule 1 (ICAM1), and MCP-1 were detected in the VE-cadherin-positive cells of aortic roots.

### Recombinant protein purification

Purification of EGFP-linked HSPB1 recombinant protein was performed by constructing a pET-SUMO vector through fusing EGFP and His-SUMO tags to the N-terminus of HSPB1, transforming Rosetta (DE3) cells, inducing expression with isopropyl β-D-1-thiogalactopyranoside, and purifying the target protein via Ni-NTA affinity chromatography.

### *In vitro* phase separation assay

*In vitro* phase separation assays were performed to evaluate the LLPS of EGFP-linked HSPB1 recombinant protein, as previously described, with modifications 42.

### Fluorescence recovery after photobleaching assay

Fluorescence recovery after photobleaching (FRAP) assays were performed to evaluate the dynamics of EGFP-linked HSPB1 *in vitro* and in HUVECs.

### Molecular docking analysis

HDRACA and HSPB1 structures were docked using the HDOCK module on the WeMol platform (https://wemol.wecomput.com/).

### Animal experiments

Low-density lipoprotein receptor null (*Ldlr^-/-^*) mice were fed a high-fat diet for 12 weeks after transduction with adeno-associated virus 9 (AAV9) carrying human HDRACA full-length (AAV-HDRACA) or negative control AAV9 (AAV-NC). Six-week-old male *Ldlr^-/-^* mice were randomly (simple randomization) assigned to two groups. The investigators were unaware of the allocation. AAV9 vectors were modified by incorporating an EC-specific TIE1 promoter sequence to enhance the specificity of target gene expression in ECs. AAV-NC and AAV-HDRACA were intravenously injected into *Ldlr^-/-^* mice. After anesthesia induction via intraperitoneal pentobarbital sodium (60 mg/kg), the mice were euthanized and carefully dissected to collect the intact aorta for further analysis, including FISH assays, immunofluorescence assays, immunohistochemistry assays, and aortic Oil Red O staining, as previously described, with modifications 17, 43-45. Following anesthesia, mice were euthanized by cervical dislocation, except for whole-mount aortic preparations, in which mice were euthanized by intracardiac perfusion with KCl and formaldehyde after anesthesia. All experiments were approved by the Institutional Animal Care and Use Committee of Sun Yat-Sen University (approval number: SYSU-IACUC-2023-000830) and were conducted in compliance with the National Institutes of Health Guide for the Care and Use of Laboratory Animals.

### Statistical analysis

Data are expressed as mean ± standard deviation (SD). Statistical analyses were performed using SPSS version 13.0 or GraphPad Prism 9.0. Data were first tested for normality. For normally distributed data, differences among groups were assessed using unpaired two-tailed Student's t-test for comparisons between two groups and one-way or two-way ANOVA followed by Tukey's multiple comparisons test for comparisons among multiple groups. Non-normally distributed data were analyzed using nonparametric tests. The Mann-Whitney *U* test was used for comparisons between two groups, and the Kruskal-Wallis test followed by Dunn's multiple comparisons test was used for comparisons among multiple groups. The chi-square test was used to compare age and smoking status between CAD patients and healthy controls. Statistical significance was defined as p < 0.05.

## Result

### HDL_healthy_ and HDL_CAD_ differentially affect adhesive and chemotactic effects of ECs by differential regulation of HDRACA expression

**Figure 1:**
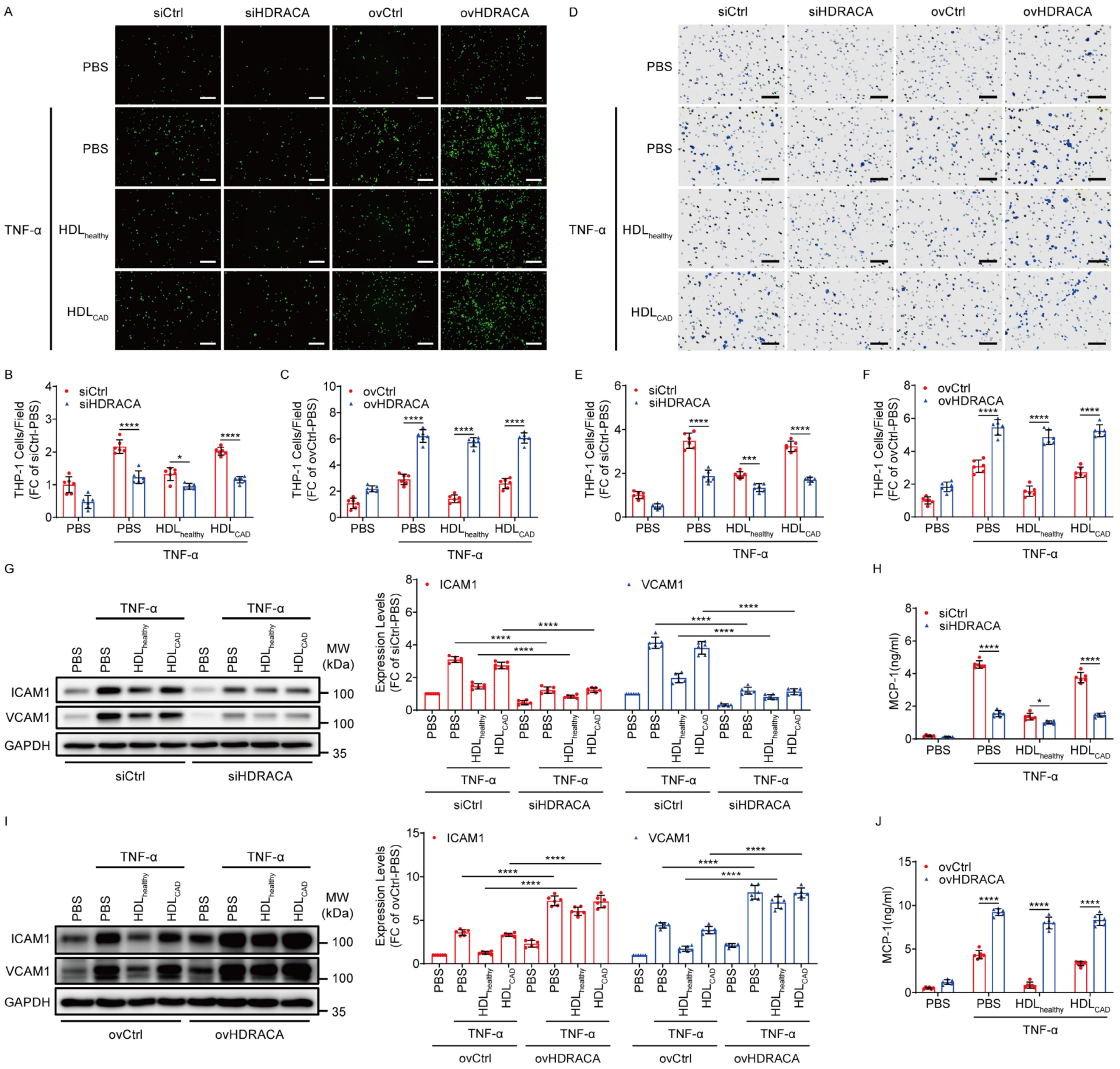
** HDL_healthy_ and HDL_CAD_ differentially affect adhesive and chemotactic effects of ECs by differential regulation of HDRACA expression.** A. Representative images of THP-1 monocytes (green) adhering to HUVECs. HUVECs were subjected to HDRACA silencing or overexpression and subsequently treated with either HDL_healthy_ or HDL_CAD_ for 24 h, followed by a 6 h incubation with TNF-α. Scale bars, 200 μm. B, C. Quantification of the adhesion assays shown in (A) for HDRACA silencing (B) or overexpression (C). D. Representative images of THP-1 monocytes (violet) migrating to the HUVEC culture medium. HUVECs were treated as described in (A). Scale bars, 100 μm. E, F. Quantification of the chemotaxis assay shown in (D) for HDRACA silencing (E) or overexpression (F). G. Representative blots (left) and quantification (right) of the western blot analysis of ICAM1 and VCAM1 in HUVECs. HUVECs were subjected to HDRACA silencing and subsequently treated with either HDL_healthy_ or HDL_CAD_ for 24 h, followed by a 6 h incubation with TNF-α. H. Quantification of ELISA analysis of MCP-1 in HUVEC culture medium. HUVECs were treated as described in (G). I. Representative blots (left) and quantification (right) of the western blot analysis of ICAM1 and VCAM1 in HUVECs. HUVECs were subjected to HDRACA overexpression and subsequently treated with either HDL_healthy_ or HDL_CAD_ for 24 h, followed by a 6 h incubation with TNF-α. J. Quantification of ELISA analysis of MCP-1 in HUVEC culture medium. HUVECs were treated as in (I). Data are presented as mean ± SD. For all experiments, n=6. Statistical significance was determined using two-way ANOVA with Tukey's multiple comparisons test (B, C, E-J). *p<0.05; ***p<0.001; ****p<0.0001.

HDL was extracted from healthy subjects and patients with CAD. HDL-bound S1P was lower in patients with CAD than in healthy individuals (Supplementary Table S1), which is consistent with our previous findings 17. The nHDLox level was significantly higher in patients with CAD than in healthy individuals, demonstrating that lipid peroxide levels in HDL_CAD_ are elevated and HDL function is impaired in CAD (Supplementary Figure S1). In our previous study, silencing of HDRACA affected the levels of genes related to the inflammatory signaling pathway for the adhesive and chemotactic effects of ECs 17. Therefore, the role of HDRACA in HDL_healthy_ and HDL_CAD_ regulating the adhesive and chemotactic effects of ECs was further investigated. Supplementary Figure S2 shows that tumor necrosis factor-α (TNF-α) did not affect HDRACA levels in HUVECs. However, HDL_healthy_ significantly downregulated HDRACA levels in TNF-α-treated HUVECs, whereas HDL_CAD_ was less effective. We silenced HDRACA by transfecting HUVECs with a lncRNA smart silencer and overexpressed HDRACA by transducing HUVECs with lentivirus carrying the full-length sequence of HDRACA, as previously described 17. HDL_healthy_ and HDRACA silencing reduced THP-1 monocyte adhesion to and migration towards HUVECs treated with TNF-α, whereas HDL_CAD_ was less effective than HDL_healthy_ (Figures 1A, B, D, and E). Moreover, overexpression of HDRACA promoted THP-1 monocyte adhesion to and migration towards HUVECs treated with TNF-α and attenuated the inhibitory effect of HDL_healthy_ (Figures 1A, C, D, and F). In addition, the expression of ICAM1, VCAM1, and MCP-1 in ECs was consistent with the effect of THP-1 monocyte adhesion to and migration towards ECs (Figures 1G-J). Taken together, these findings indicate that HDRACA is a positive regulator of the adhesive and chemotactic effects of ECs. HDL_healthy_ inhibits the adhesive and chemotactic effects of ECs by inhibiting HDRACA expression, whereas HDL_CAD_ has less of an effect in reducing HDRACA, resulting in a reduced ability to attenuate the adhesive and chemotactic effects of ECs.

### HDL_healthy_ and HDL_CAD_ differentially affect the interaction between HSPB1 and IKKβ to mediate NF-κB pathway activation by differential regulation of HDRACA expression

**Figure 2:**
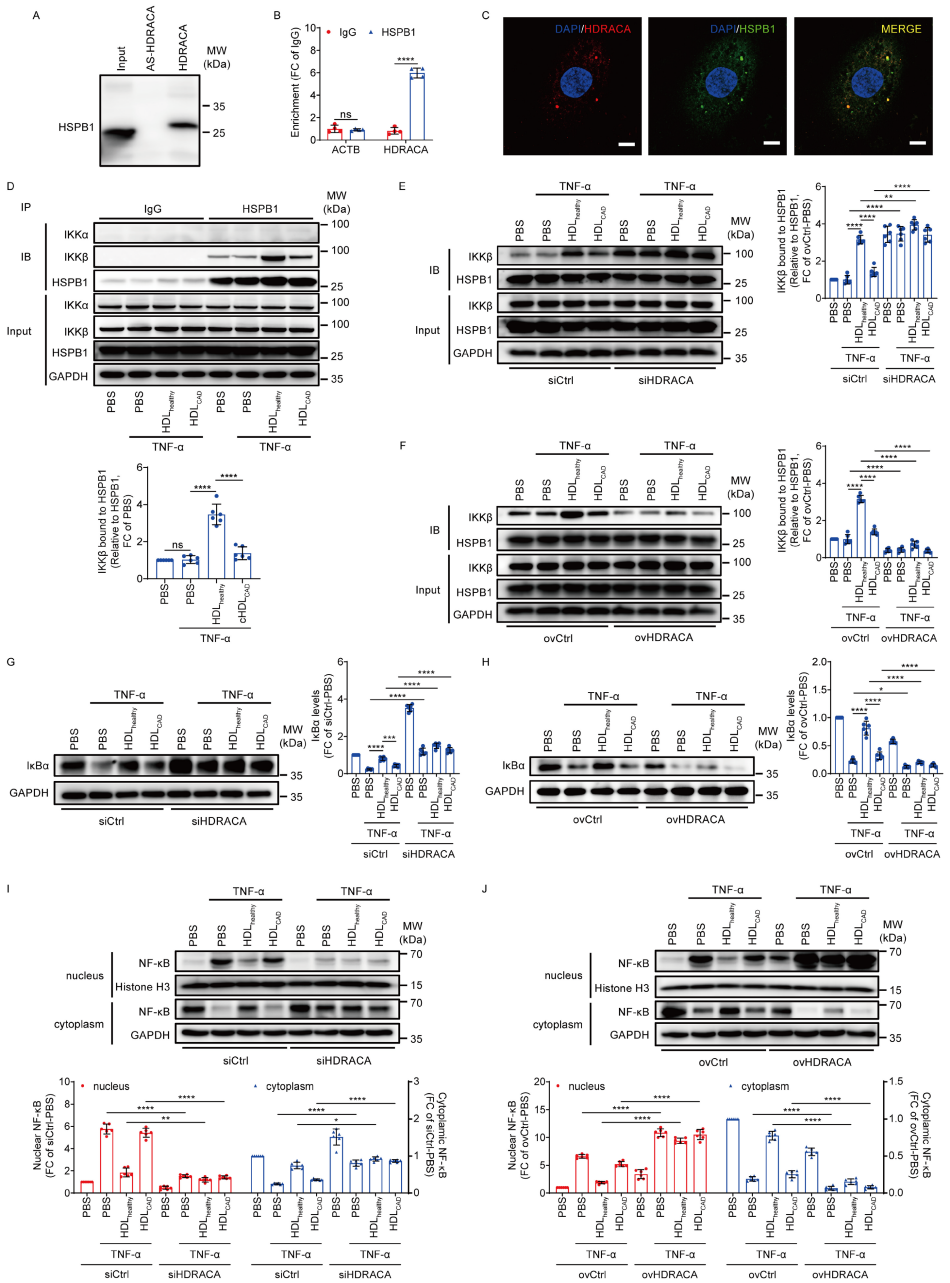
**HDL_healthy_ and HDL_CAD_ differentially affect the interaction between HSPB1 and IKKβ to mediate NF-κB pathway activation by differential regulation of HDRACA expression.** A. HSPB1 detection by western blot in protein lysates from HUVECs after RNA pull-down with biotin-labeled HDRACA or antisense control (AS-HDRACA). B. RIP assays demonstrated the interaction between HDRACA and HSPB1 in HUVECs. C. Confocal images showing the colocalization of HDRACA (red) and HSPB1 (green) in HUVECs. Nuclei were stained with DAPI (blue). Scale bars, 10 μm. D. Immunoprecipitation analysis of the interaction between HSPB1 and IKKα or IKKβ in HUVECs. HUVECs were treated with HDL_healthy_ or HDL_CAD_ for 24 h, followed by TNF-α incubation for 6 h. Representative blots (up) and quantification of IKKβ bound to HSPB1 (down) are shown. E, F. Immunoprecipitation analysis of the HSPB1/IKKβ interaction in HUVECs. HUVECs were subjected to HDRACA silencing (E) or overexpression (F), and subsequently treated with either HDL_healthy_ or HDL_CAD_ for 24 h, followed by a 6 h incubation with TNF-α. Representative blots (left) and quantification (right) are shown. G, H. Western blot analysis of IκBα in HUVECs. HUVECs were subjected to HDRACA silencing (G) or overexpression (H), and subsequently treated with either HDL_healthy_ or HDL_CAD_ for 24 h, followed by a 6 h incubation with TNF-α. Representative blots (left) and quantification (right) are shown. I, J. Western blot analysis of nuclear and cytoplasmic NF-κB in HUVECs. HUVECs were subjected to HDRACA silencing (I) or overexpression (J), and subsequently treated with either HDL_healthy_ or HDL_CAD_ for 24 h, followed by a 6 h incubation with TNF-α. Histone H3 was used as internal reference in nucleus. GAPDH was used as internal reference in cytoplasm. Representative blots (up) and quantification (down) are shown. Data are presented as mean ± SD. For A-C, n=4; For D-J, n=6. Statistical analysis was performed using unpaired Student's t-test (B), one-way ANOVA with Tukey's multiple comparisons test (D) and two-way ANOVA with Tukey's multiple comparisons test (E-J). *p<0.05; **p<0.01; ***p<0.001; ****p<0.0001; ns, not significant.

The interaction between HDRACA and HSPB1 was confirmed by RNA pull-down-western blot and RIP-RT-qPCR assays (Figures 2A and B). FISH and immunofluorescence assays showed that HDRACA and HSPB1 co-localized in the cytoplasm of HUVEC (Figure 2C). Moreover, HSPB1 knockdown promoted the expression of VCAM1, ICAM1, and MCP-1, whereas it attenuated the inhibitory effect of HDL_healthy_ (Supplementary Figure S3), suggesting that HSPB1 is an important anti-inflammatory protein involved in the anti-inflammatory activity of HDL_healthy_. However, neither HDL_healthy_ and HDL_CAD_ treatment nor silencing and overexpression of HDRACA affected the levels of HSPB1 in cultured HUVECs (Supplementary Figure S4). IKKα and IKKβ are the two catalytic subunits of IKK complex 22. We performed co-immunoprecipitation assays and found that HSPB1 bound to IKKβ in HUVECs without binding to IKKα (Figure 2D). Additionally, the interaction between HSPB1 and IKKβ in HUVECs did not change after 6 h of TNF-α treatment (Figure 2D). However, HDL_healthy_ promoted the interaction between HSPB1 and IKKβ, whereas HDL_CAD_ had less of an effect (Figure 2D). Silencing of HDRACA promoted the interaction between HSPB1 and IKKβ, whereas overexpression of HDRACA not only inhibited the interaction between HSPB1 and IKKβ but also attenuated the promoting effect of HDL_healthy_ (Figures 2E and F). Furthermore, HDL_healthy_ inhibited TNF-α-induced IκBα reduction and NF-κB nuclear translocation, whereas HDL_CAD_ had much less of an effect (Figures 2G-J). Silencing of HDRACA inhibited TNF-α-induced IκBα reduction and NF-κB nuclear translocation, whereas overexpression of HDRACA not only enhanced TNF-α-induced IκBα reduction and NF-κB nuclear translocation but also attenuated the inhibitory effect of HDL_healthy_ (Figures 2G-J). Collectively, these data suggest that HDL_healthy_ inhibits HDRACA expression to promote the interaction between HSPB1 and IKKβ, leading to IκBα upregulation and reduced NF-κB translocation into the nuclei, which reduces the transcription of NF-κB target genes. HDL_CAD_ has much less of an effect in inhibiting HDRACA expression, which cannot inhibit the transcription of NF-κB genes via the HSPB1-IKKβ-IκBα-NF-κB pathway.

### NTD and CTD of HSPB1 regulate its LLPS

**Figure 3:**
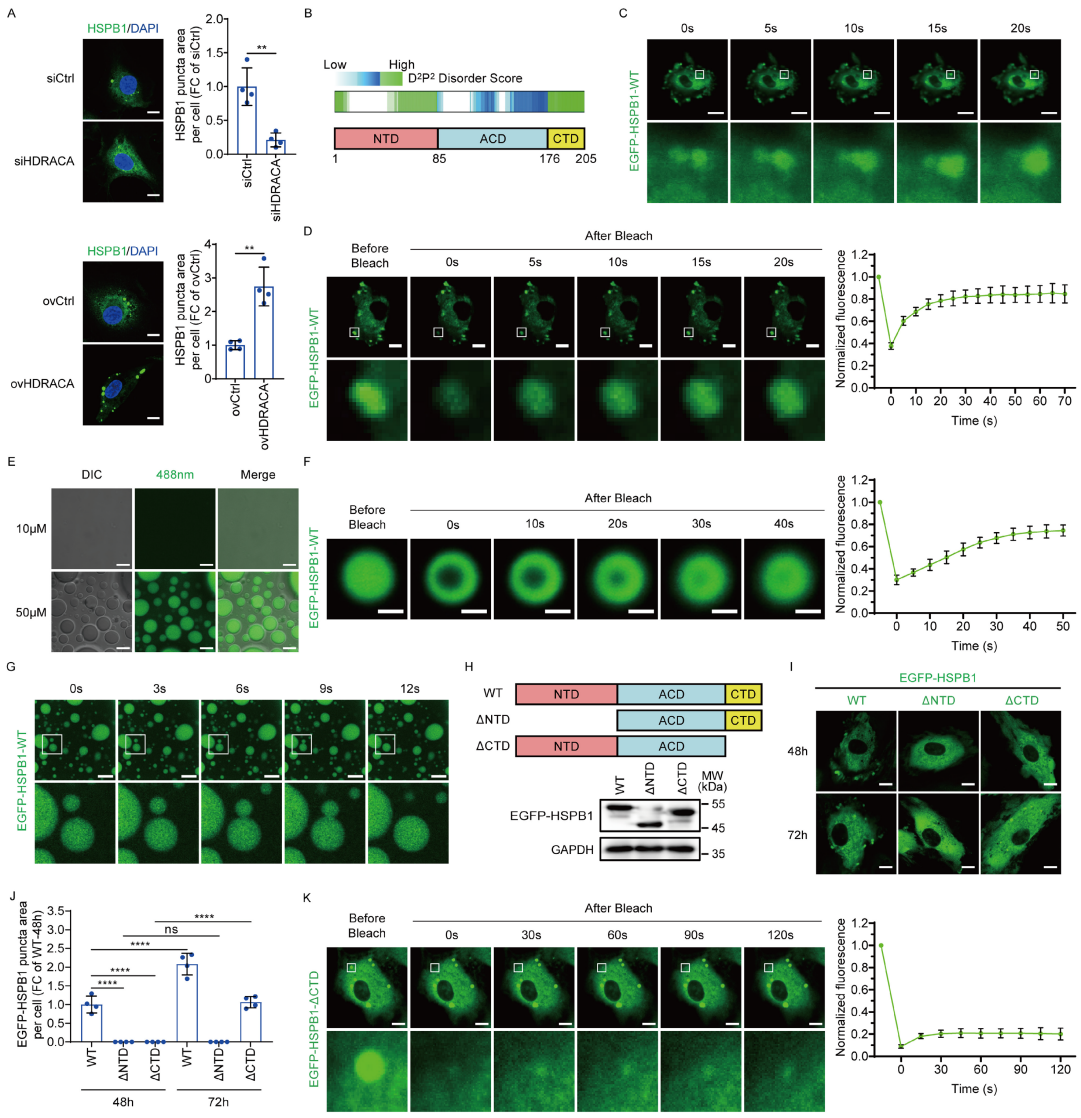
** NTD and CTD of HSPB1 regulate its LLPS.** A. Representative images (left) of HSPB1 (green) in HUVECs after silencing (up) or overexpressing (down) HDRACA. The nuclei were stained with DAPI (blue). Scale bar, 10 μm. Quantification of HSPB1 puncta area per cell is shown (right). B. Schematic illustration of human HSPB1 domains and IDR prediction using D_2_P_2_ algorithms (http://d2p2.pro/). C. Time-series images showing the fusion of EGFP-HSPB1-WT puncta in HUVECs. Scale bar, 20 μm. D. FRAP analysis of EGFP-HSPB1-WT puncta in HUVECs. Scale bar, 10 μm. Representative images (left) and quantification (right) of the fluorescence intensity recovery in the bleached region are shown. E. Representative images of *in vitro* phase separation assays for EGFP-HSPB1-WT at 10 μM and 50 μM captured using differential interference contrast (DIC) and fluorescence microscopy (λ488 nm). Scale bar, 10 μm. F. FRAP analysis of EGFP-HSPB1-WT puncta *in vitro*. Scale bar, 5 μm. Representative images (left) and quantification (right) of the fluorescence intensity recovery in the bleached region are shown. G. Time-series images showing the fusion of EGFP-HSPB1-WT puncta *in vitro*. Scale bar, 20 μm. H. Schematic illustration of EGFP-HSPB1-WT, EGFP-HSPB1-ΔNTD, and EGFP-HSPB1-ΔCTD (up). Representative western blots for EGFP-HSPB1-WT, EGFP-HSPB1-ΔNTD, and EGFP-HSPB1-ΔCTD in HUVECs 72 h post-lentiviral transduction are shown (down). I, J. Representative images of HUVECs transduced with EGFP-HSPB1-WT, EGFP-HSPB1-ΔNTD, and EGFP-HSPB1-ΔCTD for 48 and 72 h (I). Quantification of EGFP-linked HSPB1 puncta area per cell is shown (J). Scale bar, 10 μm. K. FRAP analysis of EGFP-HSPB1-ΔCTD puncta after 72 h transduction. Scale bar, 10 μm. Representative images (left) and quantification (right) of the fluorescence intensity recovery in the bleached region are shown. Data are presented as mean ± SD. For all experiments, n=4. Statistical significance was determined using unpaired Student's t-test (A) and two-way ANOVA with Tukey's multiple comparisons test (J). **p<0.01; ****p<0.0001; ns, not significant.

As shown in Figure 2C, HSPB1 aggregated to form puncta in HUVECs. Furthermore, silencing of HDRACA reduced the formation of HSPB1 puncta, whereas overexpression of HDRACA increased the formation of HSPB1 puncta (Figure 3A). The structure of HSPB1 is characterized by a central conserved α-crystallin domain (ACD) with variable N-terminal (NTD) and C-terminal domains (CTD) at its flanks 46, 47. Bioinformatics analyses of the human HSPB1 protein sequence showed that both the NTD and CTD contain several regions with multiple degrees of disorder, which are responsible for driving the LLPS of proteins (Figure 3B). LLPS is typically characterized by three features: the formation of spherical condensates, the ability of these condensates to fuse with others, and the recovery of fluorescence after photobleaching 23. We exogenously expressed EGFP-linked human HSPB1 wild-type (EGFP-HSPB1-WT) in HUVECs and found that EGFP-HSPB1-WT formed puncta and underwent fusion (Figure 3C). FRAP assays demonstrated that EGFP-HSPB1-WT puncta formation was dynamic (Figure 3D). *In vitro*, purified EGFP-HSPB1-WT protein formed droplets and fused into larger droplets over time (Figures 3E and G). The FRAP assays confirmed the dynamic properties of the droplets (Figure 3F). These results demonstrate that HSPB1 undergoes LLPS in ECs.

We further investigated the roles of the NTD and CTD in the LLPS of HSPB1. EGFP-HSPB1-WT and EGFP-linked HSPB1 mutants lacking NTD (EGFP-HSPB1-ΔNTD) or CTD (EGFP-HSPB1-ΔCTD) were expressed in HUVECs (Figure 3H). We found that EGFP-HSPB1-WT containing both the NTD and CTD formed puncta in HUVECs, with a significant increase in puncta number over time (Figures 3I and J). EGFP-HSPB1-ΔNTD containing CTD did not form puncta, whereas EGFP-HSPB1-ΔCTD containing NTD showed a delay in puncta formation (Figures 3I and J). Intriguingly, EGFP-HSPB1-ΔCTD puncta were not dynamic (Figure 3K). These data indicate that both the NTD and CTD are important domains for HSPB1 to undergo LLPS. The NTD is essential for HSPB1 aggregation initiation, whereas the CTD not only enhances aggregation but also imparts dynamic characteristics to the aggregates.

### HDRACA induces the LLPS of HSPB1 to promote the adhesive and chemotactic effects of ECs

**Figure 4:**
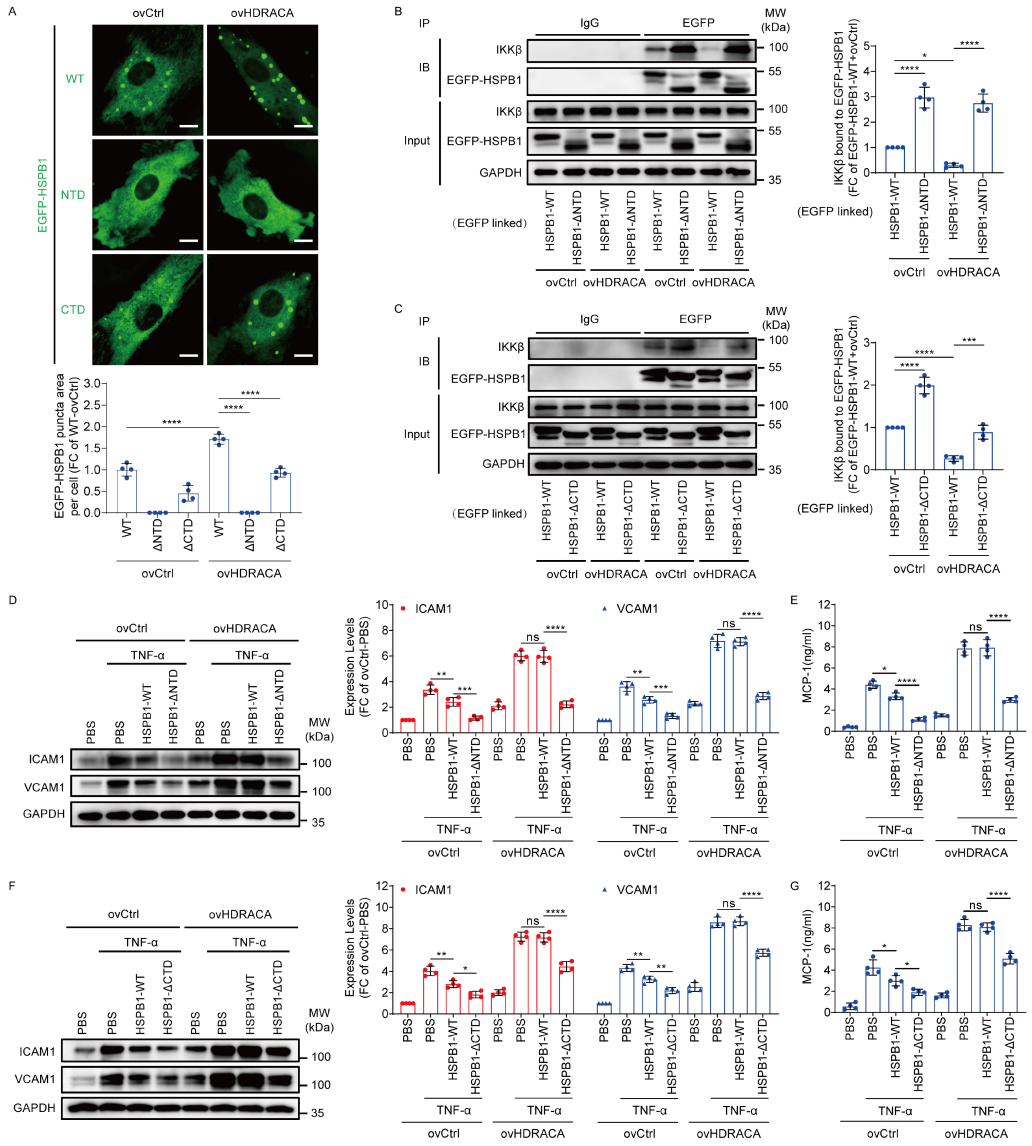
** HDRACA induces the LLPS of HSPB1 to promote the adhesive and chemotactic effects of ECs.** A. Representative images of HUVECs transduced with EGFP-linked HSPB1-WT, HSPB1-ΔNTD and HSPB1-ΔCTD for 72 h with or without HDRACA overexpression (up). Quantification of EGFP-linked HSPB1 puncta area per cell is shown (down). Scale bar, 10 μm. B. Representative blots of immunoprecipitation analysis for the interaction between EGFP-HSPB1-WT or EGFP-HSPB1-ΔNTD and IKKβ in HUVECs after overexpressing HDRACA (left). Quantification of IKKβ bound to EGFP-HSPB1-WT or EGFP-HSPB1-ΔNTD after overexpressing HDRACA (right). C. Representative blots of immunoprecipitation analysis for the interaction between EGFP-HSPB1-WT or EGFP-HSPB1-ΔCTD and IKKβ in HUVECs after overexpressing HDRACA (left). Quantification of IKKβ bound to EGFP-HSPB1-WT or EGFP-HSPB1-ΔCTD after overexpressing HDRACA (right). D. Representative blots (left) and quantification (right) of western blot analysis of ICAM1 and VCAM1 in HUVECs transduced with HSPB1-WT or HSPB1-ΔNTD after HDRACA overexpression. E. The quantification of ELISA analysis of MCP-1 from HUVECs transducing with HSPB1-WT or HSPB1-ΔNTD after HDRACA overexpression. F. Representative blots (left) and quantification (right) of western blot analysis of ICAM1 and VCAM1 in HUVECs transduced with HSPB1-WT or HSPB1-ΔCTD after HDRACA overexpression. G. The quantification of ELISA analysis of MCP-1 from HUVECs transducing with HSPB1-WT or HSPB1-ΔCTD after HDRACA overexpression. Data are presented as the mean ± SD. For all experiments, n=4. Statistical significance was determined using two-way ANOVA with Tukey's multiple comparisons test (A-G). *p<0.05; **p<0.01; ***p<0.001; ****p<0.0001; ns, not significant.

Because the NTD and CTD of HSPB1 play critical roles in LLPS, we systematically investigated the effects of HSPB1 LLPS on HDRACA-mediated HSPB1/IKKβ interaction and expression of adhesion molecules and chemokines via NTD or CTD deletion. Overexpression of HDRACA increased the formation of EGFP-HSPB1-WT puncta as endogenous HSPB1 (Figure 4A). However, deletion of the NTD significantly inhibited HDRACA overexpression-induced EGFP-HSPB1 puncta formation, whereas deletion of the CTD partially attenuated this effect (Figure 4A). Moreover, deletion of the NTD significantly inhibited HDRACA overexpression-induced suppression of HSPB1/IKKβ interaction, whereas deletion of the CTD partially attenuated this effect (Figures 4B and C). Furthermore, deletion of the NTD significantly inhibited HDRACA overexpression-induced promotion of ICAM1, VCAM1, and MCP-1 expression, whereas deletion of the CTD partially attenuated these effects (Figures 4D-G). Collectively, these data suggest that HDRACA induces LLPS of HSPB1 to inhibit the interaction between HSPB1 and IKKβ, consequently promoting the expression of adhesion molecules and chemokines in ECs.

### HDL_healthy_ and HDL_CAD_ differentially affect the phosphorylation of HSPB1 within NTD to mediate its LLPS by differential regulation of HDRACA expression

**Figure 5:**
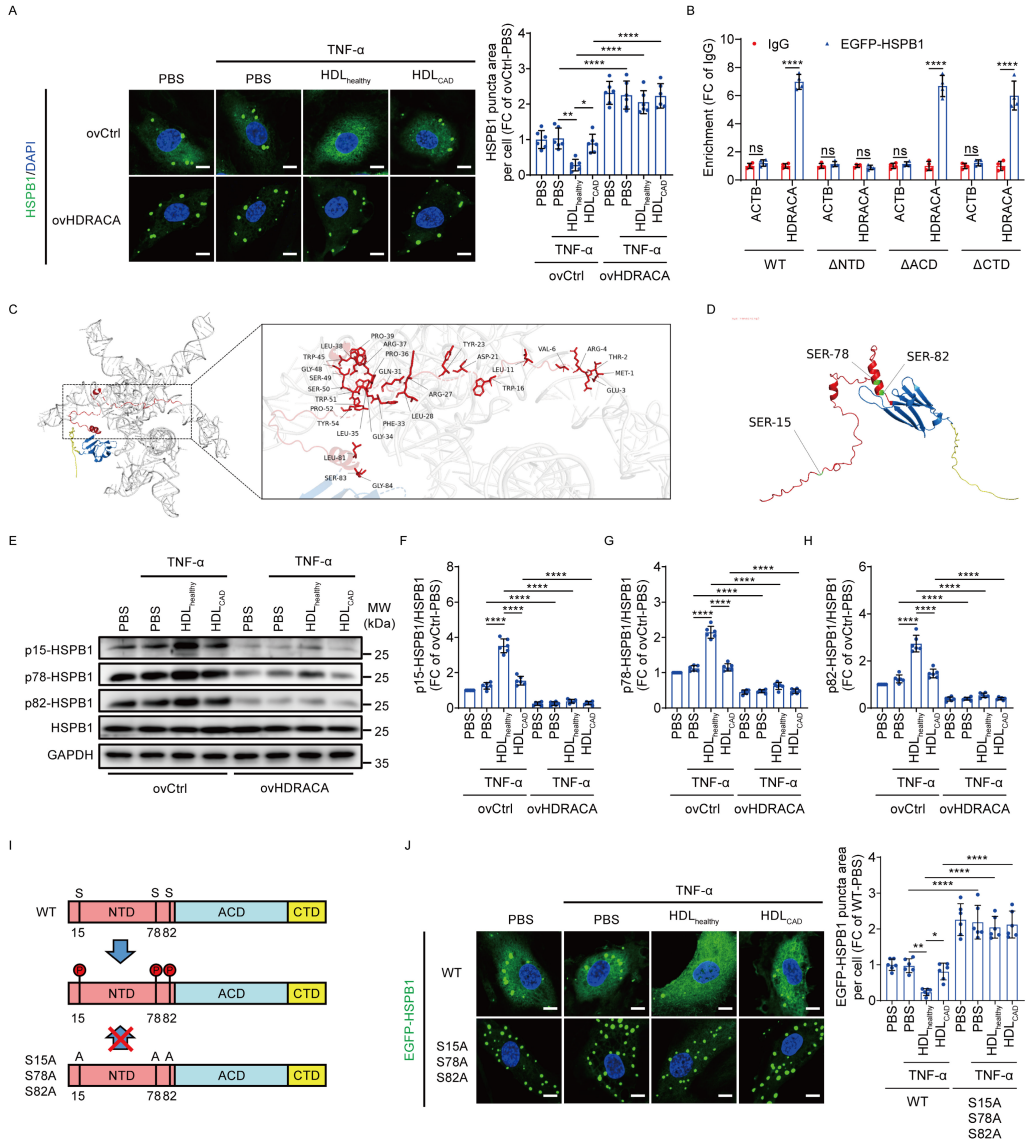
** HDL_healthy_ and HDL_CAD_ differentially affect the phosphorylation of HSPB1 within NTD to mediate its LLPS by differential regulation of HDRACA expression.** A. Representative images of HSPB1 (green) in HUVECs. HUVECs were subjected to HDRACA overexpression and subsequently treated with either HDL_healthy_ or HDL_CAD_ for 24 h, followed by a 6 h incubation with TNF-α. The nuclei were stained with DAPI (blue). Scale bar, 10 μm. Quantification of HSPB1 puncta area per cell is shown (right). B. RIP assays verified the interaction between EGFP-linked HSPB1 truncated mutants and HDRACA. C. Schematic illustration showing the molecular docking results with the highest interaction score between HDRACA and NTD of HSPB1. D. Schematic illustration showing S15, S78, and S82 in the NTD of HSPB1. E-H. Western blot analysis of p15-HSPB1, p78-HSPB1, p82-HSPB1, and HSPB1 in HUVECs. HUVECs were treated as described in (A). Representative blots (E) and quantification (F-H) are shown. I. Schematic illustration showing that mutation of serine residues at positions 15, 78, and 82 of HSPB1 to alanine can effectively block phosphorylation of these three sites. J. Representative images (left) of wild type or S15A/S78A/S82A EGFP-linked HSPB1 puncta in HUVECs. HUVECs were transduced with EGFP-linked HSPB1-WT or HSPB1- S15A/S78A/S82A, and subsequently treated with either HDL_healthy_ or HDL_CAD_ for 24 h, followed by a 6 h incubation with TNF-α. The nuclei were stained with DAPI (blue). Scale bar, 10 μm. Quantification of EGFP-linked HSPB1 puncta area per cell is shown (right). Data are presented as mean ± SD. For A, E-H, J, n=6; For B, n=4. Statistical significance was determined using two-way ANOVA with Tukey's multiple comparisons test (A, F-H, J) and unpaired Student's t-test (B). *p<0.05; **p<0.01; ****p<0.0001; ns, not significant.

Interestingly, HDL_healthy_ inhibited the formation of HSPB1 puncta, whereas HDL_CAD_ had less of an effect (Figure 5A). Additionally, the overexpression of HDRACA prompted HSPB1 puncta formation and attenuated the inhibitory effect of HDL_healthy_ (Figure 5A). Next, we investigated the effect of HDRACA in regulating the LLPS of HSPB1.We performed RIP assays after transducing EGFP-HSPB1-WT and EGFP-HSPB1 mutants into HUVECs (Figure 5B and Supplementary Figure S5). The data showed that EGFP-HSPB1-ΔNTD failed to bind to HDRACA, whereas EGFP-HSPB1-WT, EGFP-HSPB1-ΔACD, and EGFP-HSPB1-ΔCTD did (Figure 5B). This finding indicates that the NTD is necessary for the binding of HSPB1 and HDRACA in ECs. Molecular docking results showed that the NTD of HSPB1 binds to multiple sites on HDRACA via various modes (Figure 5C and Supplementary Figure S6). A previous study indicated that phosphorylation of specific N-terminal serine residues 15 (S15), 78 (S78), and 82 (S82) can inhibit HSPB1 aggregation (Figure 5D) 48. Therefore, we evaluated whether HDL_healthy_ and HDL_CAD_ regulated the phosphorylation of NTD to affect the LLPS of HSPB1 by mediating HDRACA expression. As shown in Figures 5E-H, HDL_healthy_ significantly increased the phosphorylation of S15, S78, and S82 in the NTD of HSPB1, whereas HDL_CAD_ had less of an effect. Overexpression of HDRACA decreased the phosphorylation of S15, S78, and S82 and attenuated the promoting effect of HDL_healthy_ (Figures 5E-H). A previous study indicated that mutating S15, S78, and S82 to alanine effectively inhibits phosphorylation at these sites 49. Therefore, we expressed EGFP-HSPB1-WT and EGFP-HSPB1 mutant with S15, S78, and S82 mutated to alanine (S15A/S78A/S82A) in HUVECs by lentiviral transfection (Figure 5I). HDL_healthy_ inhibited the formation of EGFP-HSPB1-WT puncta, whereas HDL_CAD_ was less effective (Figure 5J). Replacing S15, S78, and S82 with alanine significantly promoted the formation of EGFP-HSPB1 puncta and abolished the inhibitory effect of HDL_healthy_ (Figure 5J). Collectively, these data suggest that HDL_healthy_ promotes phosphorylation at N-terminal S15, S78, and S82 of HSPB1 to inhibit its LLPS by downregulating HDRACA, whereas HDL_CAD_ has less of an effect because of its inability to decrease HDRACA.

### HDRACA induces the formation of HSPB1 puncta and activates NF-κB pathway in mouse ECs

**Figure 6:**
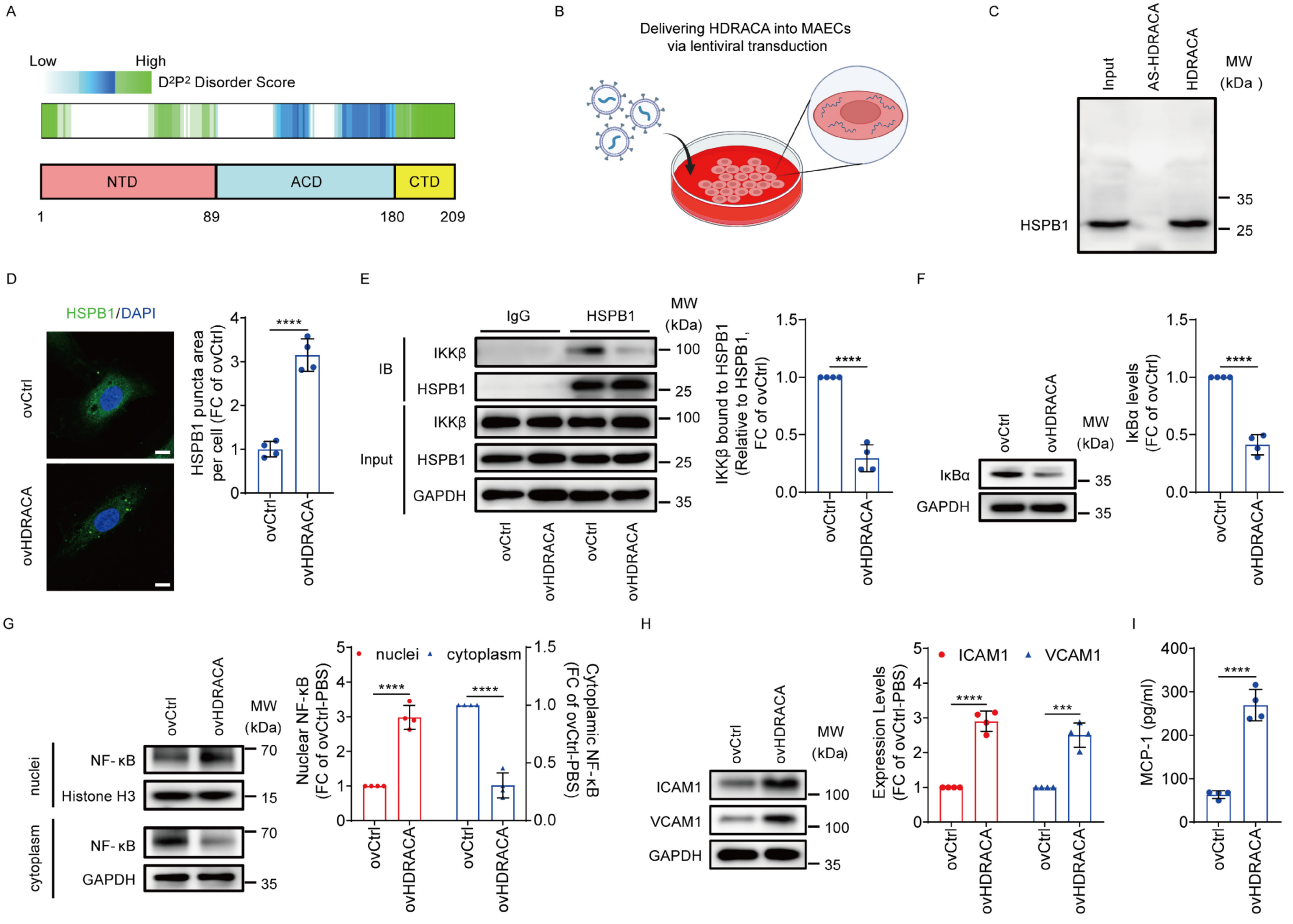
** HDRACA induces the formation of HSPB1 puncta and activates NF-κB pathway in mouse ECs.** A. Schematic illustration of mouse HSPB1 domains and IDR prediction using D_2_P_2_ algorithms (http://d2p2.pro/). B. Schematic illustration showing the ectopic expression of HDRACA in MAECs via lentiviral transduction. C. HSPB1 detection by western blot analysis in protein lysates from MAECs with HDRACA transduction subjected to RNA pull-down with biotin-labeled HDRACA or AS-HDRACA. D. Representative images of HSPB1 (green) in MAECs after transduction with HDRACA. The nuclei were stained with DAPI (blue). Scale bar, 10 μm. Quantification of HSPB1 puncta area per cell is shown (right). E. Representative blots of immunoprecipitation analysis for the interaction between HSPB1 and IKKβ in MAECs after transduction with HDRACA (left). Quantification of IKKβ bound to HSPB1 is shown (right). F. Western blot analysis of IκBα expression in MAECs after transduction with HDRACA. Representative blots (left) and quantification (right) are shown. G. Western blot analysis of NF-κB in the nuclei and cytoplasm of MAECs after transduction with HDRACA. Histone H3 was used as an internal reference for the nuclei. GAPDH was used as an internal cytoplasmic reference. Representative blots (left) and quantification (right) are shown. H. Representative blots (left) and quantification (right) of western blot analysis of ICAM1 and VCAM1 in MAECs after transduction with HDRACA. I. Quantification of ELISA analysis of MCP-1 in MAECs after transduction with HDRACA. Data are presented as mean ± SD. For C-I, n=4. Statistical significance was determined using an unpaired Student's t-test (D-I). **p<0.01; ***p<0.001; ****p<0.0001.

HDRACA is not conserved across humans and other species. Therefore, we could not directly inhibit HDRACA expression in the murine models. However, our previous study demonstrated that HDRACA interacts with evolutionarily conserved protein partners and exerts consistent functional effects on ECs across species 17. Mouse HSPB1 also contains a central ACD flanked by the NTD and CTD with multiple degrees of disorder (Figure 6A). In the present study, we ectopically expressed HDRACA in MAECs via lentiviral transduction (Figure 6B). RNA pull-down assays indicated that HDRACA bound to HSPB1 in MAECs (Figure 6C). Ectopic expression of HDRACA in MAECs promoted the formation of HSPB1 puncta (Figure 6D). Indeed, ectopic expression of HDRACA inhibited the interaction between HSPB1 and IKKβ, decreased IκBα levels, and promoted the nuclear translocation of NF-κB in MAECs (Figures 6E-G). In addition, ectopic expression of HDRACA increased ICAM1, VCAM1, and MCP-1 expression (Figures 6H and I). These data suggest that HDRACA can also induce LLPS of HSPB1 to activate the NF-κB pathway in mouse ECs, thus promoting the expression of adhesion molecules and chemokines.

### Ectopically expressing HDRACA promotes atherosclerosis

**Figure 7:**
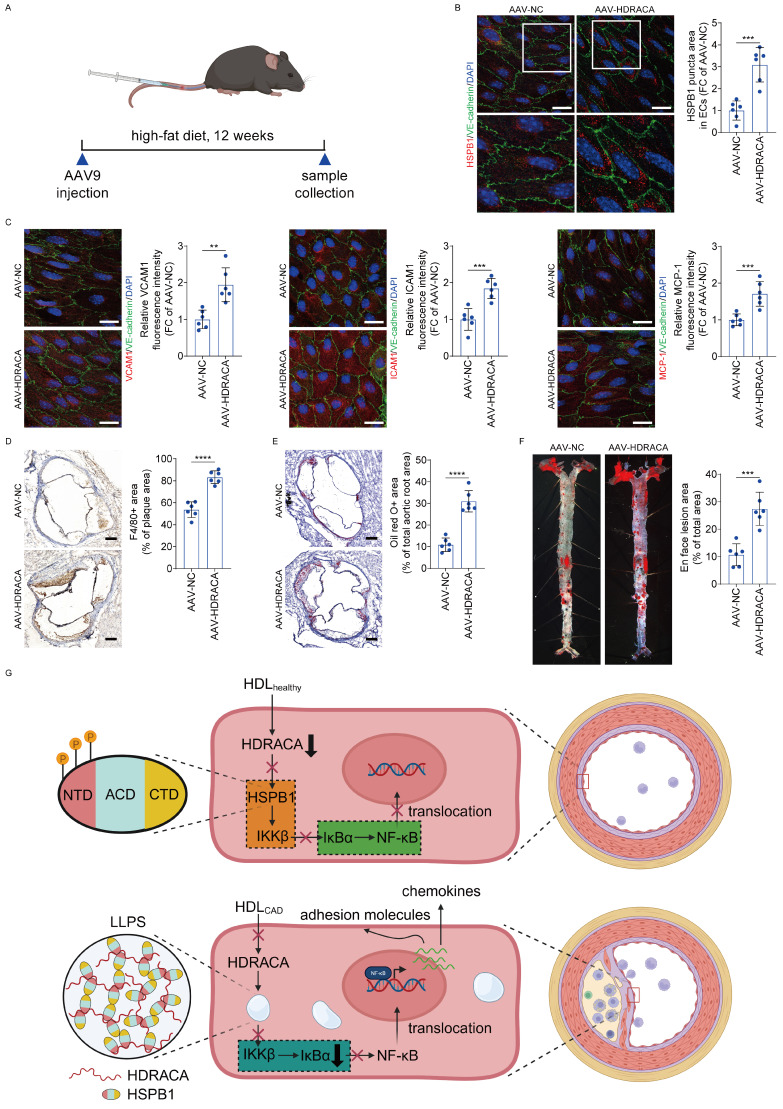
** Ectopically expressing HDRACA promotes atherosclerosis.** A. Schematic illustration of atherosclerotic model establishment in *Ldlr^-/-^* mice transduced with AAV-NC or AAV-HDRACA. B. Representative immunofluorescence staining images (left) of HSPB1 (red) in ‌ECs of whole mount en face mouse aortic preparations ‌from *Ldlr^-/-^* mice transduced with AAV-NC or AAV-HDRACA. The ECs were stained with VE-cadherin (green) and the nuclei were stained with DAPI (blue). Scale bars, 20 μm. Quantification of HSPB1 puncta area in ECs is shown (right). C. Representative immunofluorescence staining images of VCAM1 (left, red), ICAM1 (middle, red) and MCP-1 (right, red) in ECs‌ of whole mount en face mouse aortic preparations from *Ldlr^-/-^* mice transduced with AAV-NC or AAV-HDRACA. The ECs were stained with VE-cadherin (green) and the nuclei were stained with DAPI (blue). Scale bars, 20 μm. Quantitative analysis of the fluorescence intensities of VCAM1 (left), ICAM1 (middle) and MCP-1 (right) in the ECs is shown. D. Representative images (left) of immunohistochemical staining for F4/80 in aortic roots ‌from *Ldlr^-/-^* mice transduced with AAV-NC or AAV-HDRACA. Scale bars, 200 μm. Quantitative analysis of the F4/80-positive areas in the plaque regions is shown (right). E. Representative images (left) of Oil Red O staining in aortic roots‌ from *Ldlr^-/-^* mice transduced with AAV-NC or AAV-HDRACA. Scale bars, 200 μm. Quantitative analysis of the Oil Red O-positive areas in the aortic root regions is shown (right). F. Representative en face images (left) of Oil Red O staining in aorta‌ from *Ldlr^-/-^* mice transduced with AAV-NC or AAV-HDRACA. Quantitative analysis of aortic lesion areas is shown (right). G. Graphical illustration of HDL-HDRACA-HSPB1 regulatory mechanism. (1) HDL_healthy_ inhibits HDRACA expression to enhance the phosphorylation of S15, S78 and S82 in the NTD of HSPB1 in the ECs, thus promoting the interaction between HSPB1 and IKKβ, reducing the interaction between IKKβ and IκBα and increasing the interaction between IκBα and NF-κB. Consequently, reduced nuclear translocation of NF-κB attenuates the expression of downstream target genes encoding key chemokines and adhesion molecules, ultimately inhibiting immune cells infiltration and atherosclerosis formation. (2) HDL_CAD_ does not inhibit HDRACA expression in the ECs. HDRACA binds to the NTD of HSPB1 to inhibit its phosphorylation at S15, S78 and S82 sites, promoting the aggregation of HSPB1 to undergo LLPS and thus inhibit the interaction between HSPB1 and IKKβ. IKKβ binds to IκBα to induce its degradation, reducing the interaction between IκBα and NF-κB. NF-κB translocates into the nucleus to induce the expression of downstream target genes encoding key chemokines and adhesion molecules. As a result, HDL_CAD_ loses ability to inhibit immune cells infiltration and atherosclerosis formation. Data are presented as the mean ± SD. For B-F, n=6. Statistical significance was determined using unpaired Student's t-test (B-F). **p<0.01; ***p<0.001; ****p<0.0001.

Furthermore, in *Ldlr^-/-^* mice fed a high-fat diet, we investigated the effects of HDRACA delivered via AAV9 transduction on vascular inflammation and atherosclerosis formation (Figure 7A). HDRACA was successfully expressed in mouse aortic ECs (Supplementary Figure S7). Transduction of HDRACA promoted the formation of HSPB1 puncta in aortic ECs (Figure 7B). ICAM1, VCAM1, and MCP-1 levels were upregulated in ECs of the AAV-HDRACA group (Figure 7C). The number of F4/80 positive cells (predominantly macrophages and macrophage-derived foam cells) was higher in the plaques of AAV-HDRACA mice than that in AAV-NC controls (Figure 7D). Staining of aortic valve sections with Oil Red O revealed that ectopic expression of HDRACA significantly increased the Oil Red O-positive lesion area (Figure 7E). Lesions stained with Oil Red O in aortic en face preparations showed that ectopic expression of HDRACA caused a significant elevation in the lesion area compared to the AAV-NC group (Figure 7F). Collectively, these findings suggest that HDRACA promotes HSPB1 puncta formation, vascular inflammation, and atherosclerosis *in vivo*.

## Discussion

Our present study is the first to report that HDL_healthy_ downregulates the expression of HDRACA to inhibit LLPS of HSPB1 in ECs, leading to reduced NF-κB-induced expression of adhesion molecules and chemokines, which attenuates monocytes adhesion to and migration towards ECs, thereby inhibiting atherosclerosis. However, HDL_CAD_ cannot decrease the expression of HDRACA to inhibit LLPS of HSPB1 in ECs, resulting in the NF-κB-induced expression of adhesion molecules and chemokines, which stimulates the migration and adhesion of monocytes to ECs and promotes atherosclerosis.

HDL exerts multiple cardiovascular protective effects, primarily via reverse cholesterol transport, alongside anti-inflammatory, antioxidant, endothelial-protective, and pro-angiogenic activities 9, 17, 29, 50. However, several clinical studies have demonstrated that increasing HDL cholesterol levels does not reduce cardiovascular events 51-53. We and other researchers previously found that HDL undergoes compositional alterations, loses its protective ability, and becomes proinflammatory or dysfunctional in ASCVD 9, 17, 29, 54, 55 Indeed, a recent study found that HDL raised by niacin contains more atherogenic proteins 56. In addition, previous studies demonstrated that S1P levels in HDL were decreased in patients with CAD 17, 57, 58. We recently found that HDL_healthy_-bound S1P inhibits HDRACA expression to promote angiogenesis, whereas HDL_CAD_ is much less effective because of S1P deficiency 17. S1P is a bioactive lipid that can activate receptors on ECs to reduce adhesion molecules expression and attenuate inflammation 10, 59. Silencing KLF5 in EC has been shown to inhibit inflammation 60. We demonstrated that KLF5 is a transcription factor for HDRACA, and HDL-bound S1P induces ubiquitination and degradation of KLF5 through the S1P1 receptor, thereby suppressing HDRACA transcription 17. Therefore, it is possible that HDL_healthy_ may inhibit inflammation to suppress atherosclerosis through S1P-inhibited KLF5 expression to reduce the expression of HDRACA in ECs, whereas HDL_CAD_ cannot inhibit inflammation due to loss of S1P, which cannot inhibit KLF5 expression and reduce the expression of HDRACA, leading to atherosclerosis formation. Moreover, our previous study demonstrated that exogenous supplementation of S1P with HDL_CAD_ can significantly restore its inhibitory effect on HDRACA expression 17. Therefore, the level of HDL-bound S1P is a key factor in determining its inhibitory effect on HDRACA expression. Previous studies have demonstrated that the decreased S1P content in HDL_CAD_ may be associated with the oxidation of HDL 58. Our recent and present study showed increased nHDLox levels in CAD patients compared to healthy subjects 61. nHDLox represents the HDL lipid peroxide content, which can result from HDL oxidation 31. Therefore, increased nHDLox levels in CAD indicate elevated HDL oxidation, which can lead to a reduction in the S1P content in HDL_CAD_. Moreover, patients with CAD usually have disorders of lipid metabolism. Notably, fatty acids, including myristic acid, compete with S1P for binding to apolipoprotein M (ApoM) in HDL 62. These lipids may promote S1P release from HDL by competing with S1P for binding to ApoM in patients with CAD. Thus, increased nHDLox and changes in lipid composition in CAD may be critical factors for the reduced S1P levels in HDL_CAD_, which attenuates the inhibitory effect of HDL_CAD_ on HDRACA expression. Collectively, this is the first novel finding that HDL_healthy_ inhibits inflammation and atherosclerosis by suppressing HDRACA expression, whereas HDL_CAD_ loses its atheroprotective ability owing to its reduced ability to inhibit HDRACA.

Recent studies reported that LLPS can mediate vascular inflammation and atherosclerosis formation 24, 25. Additionally, LLPS plays a crucial role in regulating various endothelial functions 24, 63, 64. Importantly, lncRNAs may be important drivers of LLPS 26, 27. lncRNAs can initiate condensate formation by binding to RNA-binding proteins to form ribonucleoprotein particles, which subsequently recruit additional non-condensed proteins through intrinsically disordered regions (IDRs) to drive LLPS 26, 27. Previous studies demonstrated that lncRNAs can efficiently regulate protein activities by recruiting far outnumbered protein molecules to undergo LLPS 26, 27. LLPS is recognized as a regulatory mechanism that can enrich specific factors while excluding others, thereby coordinating the specificity and efficiency of signal transduction 23. The present study demonstrates that HDRACA can induce LLPS of HSPB1, which sequesters the interaction between HSPB1 and IKKβ and activates the NF-κB pathway. Additionally, other studies have reported that HSPB1 can co-phase separate with TDP-43 and FUS to regulate amyloid fibril aggregation of these proteins 46, 47. Therefore, HSPB1 may form compartmentalized reaction hubs through LLPS to bind to specific molecules and exert distinct regulatory functions.

Small heat shock proteins, including HSPB1, act as molecular chaperones that regulate protein folding and aggregation. Previous studies have suggested that the ACD of HSPB1 is the major active domain for its chaperone activity, whereas the NTD and CTD modulate this activity 65-67. Additionally, HSPB1 can aggregate to form oligomers, which reduce its affinity for substrates 68-70. Previous studies have shown that phosphorylation within the NTD of HSPB1 at serine residues 15, 78, and 82 decreases oligomerization and promotes interaction with IKKβ, thereby limiting NF-κB activation 49, 68-70. The present study further demonstrates that inhibiting the phosphorylation of serine residues 15, 78, and 82 in the NTD of HSPB1 promotes its LLPS. Moreover, a recent study reported that CCF0054500 can inhibit the phosphorylation of HSPB1 at serine residues 15, 78, and 82 in platelets, thereby disrupting the actin cytoskeleton and reducing platelet aggregation 71. Thus, LLPS-mediated compartmentalization of HSPB1 may be critical for cytoskeletal maintenance. This requires further investigation into the functions of HSPB1 LLPS-formed reaction hubs in the future.

Our present study found that HDRACA inhibits the phosphorylation of HSPB1 at S15, S78, and S82 sites of the NTD, likely through multiple binding interactions with the NTD. The direct binding of HDRACA and HSPB1 may create steric hindrance, physically blocking upstream kinases, including MAPKAPK2/3, from accessing serine residues 72. Additionally, HDRACA promotes LLPS of HSPB1 into biomolecular condensates, which may alter the conformational accessibility of its NTD, further limiting kinase accessibility. Thus, HDRACA may suppress the phosphorylation of HSPB1 through a combination of binding-induced blockade and promotion of a phase-separated state. HDRACA may serve as a scaffold that promotes the interaction and aggregation of multiple HSPB1 monomers, thereby forming an HDRACA-HSPB1 complex that serves as a core for driving LLPS. Moreover, the CTD of HSPB1 within the complex can enhance the formation of LLPS condensates via weak multivalent interactions with free HSPB1 or HSPB1 from the other complexes. Interestingly, the absence of the CTD causes HSPB1 condensates to lose their dynamic liquid-like state, potentially leading to amyloid aggregation, insoluble aggregation, or liquid-solid phase separation 73-75. This is the second novel finding that HDRACA induces vascular inflammation to promote atherosclerosis by increasing LLPS of HSPB1 to activate the NF-κB pathway.

An important finding is that inhibition of HSPB1 aggregation may be a critical therapeutic step to reduce vascular inflammation and atherosclerosis. Therefore, the direct targeting of HSPB1 is a potential strategy for inhibiting atherosclerosis. Furthermore, we not only found that deletion of the NTD more effectively reduced the formation of HSPB1 condensates than deletion of the CTD but also demonstrated that HDRACA primarily targets the NTD of HSPB1. Therefore, the development of NTD-targeted therapeutic agents to inhibit HSPB1 aggregation may offer a promising strategy for inhibiting atherosclerosis. Currently, technologies, including virtual screening and surface plasmon resonance, facilitate high-throughput screening of large chemical libraries, providing a strong foundation for the development of NTD-targeted drugs. Alternatively, improving HDL function or inhibiting HDRACA represent two other promising strategies. These strategies can not only attenuate atherosclerosis but also promote angiogenesis, facilitating tissue revascularization following atherosclerotic stenosis.

This study had several limitations. First, although HUVECs are widely used in atherosclerosis research because of their accessibility and high proliferative capacity, our findings require further validation in human artery endothelial cells, which are more directly relevant to atherosclerosis. Second, although our study supports a functional role for HDRACA in promoting atherosclerosis, we acknowledge that direct inhibition of HDRACA is essential to conclusively establish its role as a key therapeutic target. We recently made significant findings regarding this matter. The human HDRACA-encoding gene (*LOC105373383*) is located on the sense strand of the X chromosome between the *ATP2B3* and *DUSP9* genes. We identified an expressed sequence tag, *CF424538*, located on the antisense strand of the mouse X chromosome between the *Atp2b3* and *Dusp9* genes. Although *CF424538* shares only 45.8% homology with human HDRACA, Mfold analysis showed that their secondary structures were highly similar (data not shown). Since HDRACA primarily regulates substrate protein function via conformational binding, we hypothesized that *CF424538* may be a potential structural homolog candidate for HDRACA 76. Next, we will investigate whether *CF424538* binds to murine HSPB1 and then evaluate atherosclerosis development following *CF424538* silencing in high-fat diet-fed *Ldlr*^-/-^ mice. Moreover, advanced structural validation, including comparative analysis of RNA three-dimensional structures should be performed to confirm the structural consistency between HDRACA and *CF424538*. Additionally, three-dimensional organoid or artery-on-a-chip models derived from human cells provide a promising platform for investigating the effect of HDRACA silencing on atherosclerosis formation 77-79.

Overall, this study highlights that HDRACA plays a critical regulatory role in HDL-mediated vascular inflammation and atherosclerosis. The differential regulation of HDRACA by HDL_healthy_ and HDL_CAD_ may explain their distinct anti-inflammatory effects. Furthermore, our findings directly demonstrate that HDRACA promotes atherosclerosis formation, positioning it as a key link between HDL dysfunction and atherosclerosis development. Additionally, this study uncovered an important role of lncRNA-regulated LLPS in vascular inflammation, offering a novel perspective for exploring the mechanisms of atherosclerosis. We proposed three potential anti-atherosclerosis strategies: improving HDL function, inhibiting HDRACA, and blocking the LLPS of HSPB1. The clinical application of these strategies holds great promise for advancing ASCVD therapy.

## Supplementary Material

Supplementary methods, figures and tables.

## Data Availability

The authors will provide the data supporting the findings of this study upon reasonable request to corresponding authors.
