# Supplementary material for: High density-lipoprotein regulates liquid-liquid phase separation of heat shock protein β-1 by lncRNA HDRACA to affect vascular inflammation and atherosclerosis
Source: Int J Biol Sci. 2026 Jul 1;22(12):6521–38. doi: 10.7150/ijbs.135145 (PMC13411970; doi:10.7150/ijbs.135145)
Supplement: Supplementary file 1 — Supplementary methods, figures and tables. [file ijbsv22p6521s1.pdf]

Supplementary Materials

High density-lipoprotein regulates liquid-liquid phase separation of heat shock protein β-1 by lncRNA HDRACA to affect vascular inflammation and atherosclerosis

Zhi-Wei Mo, MD,PhD[#], Yi-Fang Liu, BS[#], Yi-Xin Zhang, MD,PhD[#], Yan Li, PhD[#], Yang Cao, MD[#], Chu-Yu Liu, MD,PhD[#], Le Li, BS, Yue-Ting Kang, MD, Zhen-Sheng Ma, MD, Yue-Ming Peng, MD,PhD, Zhi-Jun Ou, MD,PhD[*], Guang-Qi Chang, MD,PhD[*], Jing-Song Ou, MD,PhD[*]

*Correspondence to

Z.J.O: zhijunou@163.com

G.Q.C: changgq@mail.sysu.edu.cn

J.S.O: oujs@mail.sysu.edu.cn, oujs2000@163.com

This file includes:

Expanded Materials and Methods

Supplementary Tables S1 to S3

Supplementary Figures S1 to S7

Expanded Materials and Methods

Study populations and sample acquisition

Age- and sex-matched patients with coronary artery disease (CAD) and healthy individuals without cardiovascular risk factors were enrolled to isolate high-density lipoprotein (HDL). Patients with CAD were included based on coronary angiography showing stenosis ≥70% and excluded if they had an accompanying infection, other inflammatory or autoimmune diseases, advanced renal or hepatic failure, neoplastic diseases, or a history of major surgery or trauma within the previous month. To avoid medication interference, the enrolled patients with CAD had not received any drug treatment in the three months prior to HDL isolation. Detailed information is provided in the Supplementary Table S1. Quantification of HDL-bound sphingosine 1-phosphate (S1P) was performed using liquid chromatography-tandem mass spectrometry (LC-MS/MS) analysis, as previously described[1]. Written informed consent was obtained from all the participants. Plasma was isolated from venous blood and subsequently used for HDL isolation or stored at -80 °C. The study protocol was approved by the Ethics Review Board of the First Affiliated Hospital, Sun Yat-sen University (Approval number: [2023]374) and was conducted in accordance with the principles outlined in the Declaration of Helsinki.

HDL isolation

HDL was isolated from individual plasma samples by sequential ultracentrifugation as described previously[1-4]. The isolated HDL was stored at 4 °C and used within 3 weeks.

Assessment of nHDLox

HDL lipid peroxide content (HDLox) was measured and normalized (nHDLox) by HDL-C levels and the mean value of a pooled serum control from healthy subjects as previously described[5].

Cell culture

Human umbilical vein endothelial cells (HUVECs, Cat. 8000, USA, ScienCell), mouse aortic endothelial cells (MAEC, Cat. CP-M075, China, Procell) and THP-1 monocytes (Cat. THP-1, China, Procell) were purchased and cultured at 37 °C in a humidified incubator with 5% CO_2. HUVECs and MAECs were cultured in endothelial cell medium (ECM, Cat.1001, USA, ScienCell) containing 5% fetal bovine serum (FBS, Cat.0025, USA, ScienCell), 1% endothelial

cell growth supplement (ECGS, Cat.1052, USA, ScienCell) and 1% penicillin/streptomycin (P/S, Cat.0503, USA, ScienCell). THP-1 monocytes were cultured in RPMI Medium 1640 (Cat. C11875500BT, USA, Gibco) containing 10% FBS and 1% P/S. HUVECs were cultured in ECM containing 0.5% FBS overnight prior to stimulation with 100 μg protein/ml $HDL_{healthy}$ or HDL_{CAD}, followed by incubation with 100 ng/ml tumor necrosis factor-α (TNF-α) for 6 h. For functional and molecular biological assays involving HDL, HDL preparations were randomly chosen from the HDL sample pool.

Adhesion assay

Calcein AM (Cat. 206700, USA, Millipore Sigma) was diluted to a final concentration of 1 μM in Hanks' balanced salt solution (HBSS, Cat. 14025092, USA, Gibco) containing 2 mM Ca^{2+}, 2 mM Mg^{2+}, and 2 mM HEPES (Ca^{2+}/Mg^{2+} HBSS). Then, THP-1 cells were resuspended in the prepared Calcein AM solution and incubated at 37 °C in a humidified incubator with 5% CO_2 for 30 min. After incubation, cells were washed twice with Ca^{2+}/Mg^{2+} HBSS to remove excess dye and resuspended in the same buffer to a final concentration of 1.5×10^6 cells/mL. Then, HUVECs were prepared by aspirating their culture medium and replacing it with Calcein AM-labeled THP-1 cell suspension. The co-culture was incubated at 37 °C in a humidified incubator with 5% CO_2 for 30 min. Unbound THP-1 cells were removed by aspiration, and HUVEC monolayers were washed twice with Ca^{2+}/Mg^{2+} HBSS to remove residual non-adherent cells. Adherent cells were fixed with 4% paraformaldehyde for 10 min at room temperature. Fluorescent images were acquired using a fluorescence microscope (DMi8, Leica, Germany) and the number of adherent THP-1 cells per field was quantified.

Chemotaxis assay

THP-1 monocytes were resuspended in serum-free RPMI 1640 medium. Conditioned medium from HUVECs treated according to the experimental design was collected for use as a chemoattractant. Transwell inserts with 8.0 μm pore size PET track-etched membranes (Cat. 353097, USA, Falcon) were placed in a 24-well plate. The resuspended THP-1 cells were seeded into the upper chamber of the inserts at a density of 1×10^5 cells/mL, and the collected HUVEC-conditioned medium was added to the lower chamber. The plates were then incubated at 37 °C in a humidified incubator with 5% CO_2 for 12 h. After incubation, non-migrated cells on the upper surface of the membrane were gently scraped off using a cotton swab. After being

fixed with 4% paraformaldehyde, the migrant cells on the lower surface of the membrane were stained with crystal violet and counted under a light microscope (DMi1, Leica, Germany).

RNAi and antisense silencing studies

The lncRNA Smart Silencer and small interfering RNAs (siRNAs) were designed and synthesized by RiboBio, China to silence lncRNA *high-density lipoprotein-regulated angiogenesis in coronary artery disease* (HDRACA) or knock down heat shock protein β-1 (HSPB1). The lncRNA Smart Silencer negative control and siRNA negative control were used as the negative controls. Cells were transfected with lncRNA Smart Silencers (200 nM) or siRNAs (100 nM) using the Lipofectamine RNAiMAX Transfection Reagent (Cat. 13778030, USA, Thermo Fisher Scientific) according to the manufacturer's instructions. The target sequences of the lncRNA Smart Silencers and siRNAs are listed in the Supplementary Table S2.

Lentiviral transduction

All lentiviruses were designed and purchased from Umine-biology, China. To reduce potential interference between the enhanced green fluorescent protein (EGFP) and wild-type or mutant HSPB1 domains, a linker was inserted between them to maintain their independent functions[6]. Lentiviral transduction was performed as follows. The cells were cultured until they reached 30-50% confluence. Subsequently, the cells were exposed to lentiviral particles at a multiplicity of infection (MOI) of 5. Unless otherwise specified, the infected cells were cultured for an additional 72 h before further experiments.

RNA extraction, reverse transcription, and RT-qPCR

Total RNA was extracted using Trizol Reagent (Cat. T9424, USA, Sigma-Aldrich) and reverse transcribed into complementary DNA (cDNA) using Transcriptor cDNA Synth. kit (Cat. 4897030001, Switzerland, Roche) according to the manufacturer's instructions. Real-time quantitative polymerase chain reaction (RT-qPCR) was performed in a Bio-rad CFX96 system as previously described[1]. Primers used for RT-qPCR are listed in the Supplementary Table S3. All results were normalized to the endogenous reference, glyceraldehyde-3-phosphate dehydrogenase (GAPDH).

Western blot

Western blot analysis was performed as previously described[7-10]. Briefly, lysed cells were

centrifuged to collect the supernatant. The proteins were quantified using a bicinchoninic acid protein assay (Cat. 23225, USA, Thermo Fisher Scientific) and mixed with sodium dodecyl sulfate-polyacrylamide gel electrophoresis (SDS-PAGE) loading buffer (Cat. P0015F, China, Beyotime). Equal amounts of protein were separated by SDS-PAGE and transferred to polyvinylidene fluoride membranes (Cat. 03010040001, Switzerland, Roche). After being blocked with Tris-buffered saline/Tween-20, the membranes were probed with primary antibodies at 4 °C overnight and then incubated with secondary antibodies at room temperature for 1 h. Finally, immunoreactive bands were visualized using ECL (AI600, GE Healthcare, USA). The primary antibodies used in this study were against intercellular adhesion molecule 1 (ICAM1, 1:1000, Cat. 67836, USA, Cell Signaling Technology or 1:1000, Cat. ab222736, UK, Abcam), vascular cell adhesion molecule 1 (VCAM1, 1:1000, Cat. 13662, USA, Cell Signaling Technology or 1:2000, Cat. ab134047, UK, Abcam), GAPDH (1:1000, Cat. 60004-1-Ig, USA, Proteintech), inhibitor of kappa-B kinase α (IKKα, 1:1000, Cat. 11930, USA, Cell Signaling Technology), inhibitor of kappa-B kinase β (IKKβ, 1:1000, Cat. 8943, USA, Cell Signaling Technology), HSPB1 (1:1000, Cat. 95357, USA, Cell Signaling Technology or 1:1000, Cat. MA5-32473, USA, Invitrogen), Phospho-HSPB1 (Ser15, 1:1000, Cat. AF3080, USA, Affinity Biosciences), Phospho-HSPB1 (Ser78, 1:1000, Cat. AF3081, USA, Affinity Biosciences), Phospho-HSPB1 (Ser82, 1:1000, Cat. 9709, USA, Cell Signaling Technology), inhibitor of κB α (IκBα, 1:1000, Cat. 4814, USA, Cell Signaling Technology), nuclear factor-κB (NF-κB, 1:1000, Cat. 8242, USA, Cell Signaling Technology), Histone H3 (1:1000, Cat. 4499, USA, Cell Signaling Technology), and EGFP (1:1000, Cat. sc-9996, USA, Santa Cruz Biotechnology).

Enzyme-linked immunosorbent assay

The levels of monocyte chemoattractant protein-1 (MCP-1) in the culture medium of HUVECs and MAECs were measured using enzyme-linked immunosorbent assays (ELISA). Human MCP-1 levels were quantified using a Human MCP-1 ELISA Kit (Cat. CSB-E04655h, China, CUSABIO), whereas mouse MCP-1 levels were determined using a Mouse MCP-1 ELISA Kit (Cat. CSB-E07430m, China, CUSABIO). All procedures were performed according to the manufacturer's instructions.

Immunoprecipitation

Immunoprecipitation assays were performed as described previously [1, 11, 12]. Antibodies or IgG were incubated with Protein A/G magnetic beads (Cat. B23201, USA, bimake) for 30 min at room temperature. The cells were lysed and centrifuged. The protein supernatant was added to the antibody-magnetic bead complexes and incubated overnight at 4 °C. After the beads were collected and washed, SDS-PAGE loading buffer was added, and the immunocomplexes were heated for 5 min at 95 °C. Finally, denatured proteins were subjected to western blot analysis. The antibodies against HSPB1 (Cat. 95357, USA, Cell Signaling Technology) and EGFP (Cat. sc-9996, USA, Santa Cruz Biotechnology) was used in this study.

Nuclear/cytoplasmic fractionation

Nuclear and cytoplasmic fractions were isolated using NE-PER Nuclear and Cytoplasmic Extraction Reagent (Cat. 78833, USA, Thermo Fisher Scientific) according to the manufacturer's instructions.

RNA pull-down assay

RNA pull-down assays were performed using the Pierce™ Magnetic RNA-Protein Pull-Down Kit according to the manufacturer's instructions, as described previously[1]. Briefly, streptavidin magnetic beads were mixed with biotinylated RNA probes and incubated at room temperature for 30 min. Subsequently, RNA-coupled beads were incubated with the cell lysate at room temperature overnight. The RNA-binding protein complexes were eluted and denatured by boiling for 10 min. Finally, western blot analysis was performed to identify the retrieved proteins.

RNA immunoprecipitation

RNA immunoprecipitation (RIP) assays were performed using the Magna RIP RNA-Binding Protein Immunoprecipitation Kit (Cat. 17700, USA, Millipore Sigma) according to the manufacturer's protocol, as described previously[13, 14]. Briefly, antibodies or IgG were incubated with Protein A/G magnetic beads at room temperature for 30 min. Then, cell lysates were added to bead-antibody complexes and incubated overnight at 4 °C. Total RNA was extracted from the immunoprecipitated material and analyzed using RT-qPCR. The antibodies used in this study were against HSPB1 (1:200, Cat. sc-13132, USA, Santa Cruz Biotechnology) and EGFP (1:200, Cat. sc-9996, USA, Santa Cruz Biotechnology).

Fluorescence in situ hybridization and immunofluorescence assays in cells

Fluorescence in situ hybridization (FISH) assays were performed to detect HDRACA expression in HUVECs using the Ribo™ Fluorescent In Situ Hybridization Kit according to the manufacturer's instructions. Briefly, after being fixed with RNAase-free 4% paraformaldehyde and permeabilized in 0.5% TritonX-100, cells were hybridized with cyanine 3 (Cy3)-labeled HDRACA detection probes (customized by RiboBio) at 53 °C overnight. The cells were subjected to immunofluorescence staining for HSPB1. Finally, the nuclei of the cells were counterstained with 4',6-diamidino-2-phenylindole (DAPI) and imaged using a confocal laser scanning microscope equipped with a core data acquisition system (LSM880, Zeiss, Germany).

For immunofluorescence staining of HUVECs and MAECs, the cells were fixed with 4% paraformaldehyde for 15 min. After being blocked and permeabilized with phosphate-buffered saline (PBS) containing 5% BSA, 5% normal goat serum, and 0.3% Triton X-100 for 1 h, the cells were incubated with HSPB1 Antibody (1:500, Cat. 95357, USA, Cell Signaling Technology) overnight at 4 °C, followed by 1 h of incubation with Alexa Fluor 488-conjugated secondary antibody (Cat. A-11008, USA, Invitrogen) at room temperature. Finally, the nuclei were counterstained with DAPI, and images were captured using a confocal laser scanning microscope equipped with a core data acquisition system (LSM880, Zeiss, Germany).

Recombinant protein purification

To purify the target proteins, fusion constructs were generated by attaching EGFP and a hexahistidine-tagged small ubiquitin-like modifier (His-SUMO) to the N-terminus of HSPB1. cDNA sequences encoding these fusion proteins were cloned into the pET-SUMO vector (GENE CREATE, China).

For protein production, the fusion plasmids were transformed into Rosetta (DE3) cells, followed by the selection of kanamycin-resistant colonies. The colonies were cultured in Luria-Bertani (LB) medium containing 50 μg/ml kanamycin (Cat. 0408, USA, Amresco) and incubated at 37 °C with agitation until the optical density at 600 nm (OD600) reached 0.6. Protein expression was initiated by addition of isopropyl β-D-1-thiogalactopyranoside (Cat. 0487, USA, Amresco) to a final concentration of 0.1 mM, and the cultures were incubated at 16 °C for 12 h. Cells were harvested by centrifugation at 8000 rpm for 3 min, then resuspended in lysis buffer (20 mMTris base, 500 mM NaCl, and 10% glycerol, 0.1 mg/ml lysozyme, pH

8.0). The cell suspension was lysed by sonication on ice, and the cell debris was removed by centrifugation at 16000 rpm for 50 min at 4 °C. The supernatant was collected and applied to pre-equilibrated Ni-NTA agarose columns (Cat. R90101, USA, Invitrogen) that had been equilibrated with lysis buffer. The columns were washed with wash buffer (20 mM Tris base, 500 mM NaCl, and 10% glycerol, pH 8.0), and the fusion proteins were eluted with imidazole at concentrations of 20 mM, 60 mM, 200 mM, and 500 mM. The elution fractions were collected in successive aliquots until no further color change was observed in the G250 detection solution. The eluted fractions were analyzed by SDS-PAGE. The purified fusion proteins were dialyzed against PBS and stored at -80 °C until further use.

In vitro phase separation assay

In vitro phase separation assays were performed as previously described with modifications [15]. Briefly, the phase separation assay was performed in a physiological buffer (15 mM NaCl, 130 mM KCl, 1.5 mM $MgCl_2$, 5 mM KH_2PO_4, 20 mM Tris-HCl, pH 7.5, and 1 mg/mL BSA) containing both specified protein concentrations and 10% (w/v) PEG8000. These assays were performed in glass-bottomed dishes (NEST), which were sealed with an optically clear adhesive film to minimize evaporation. The phase separation process was visualized using a laser scanning microscope equipped with a core data acquisition system (LSM880, Zeiss, Germany).

Fluorescence recovery after photobleaching assay

Fluorescence recovery after photobleaching (FRAP) assays were performed using a confocal laser scanning microscope equipped with a 488-nm laser (LSM880, Zeiss, Germany). For the in vitro experiments, a bleach spot was placed at the center of a droplet, and sequential images were captured at 1-second intervals to monitor the fluorescence recovery within the droplet. For in vivo experiments, droplets were photobleached using a 488-nm laser pulse. The recovery from photobleaching was recorded at the indicated times. The prebleach fluorescence intensity was standardized to a value of 1, ensuring consistency in the analysis of recovery dynamics.

Molecular docking analysis

The secondary structure of HDRACA with the lowest free energy predicted using mfold (http://www.unafold.org/) was subjected to the 3dRNA/DNA Web Server (http://biophy.hust.edu.cn/new/) to predict and model the three-dimensional structure. The

molecular structure of the full-length human HSPB1 was predicted using AlphaFold 3.0 (https://alphafoldserver.com/). HDRACA and HSPB1 structures were docked using the HDOCK module on the WeMol platform (https://wemol.wecomput.com/). The conformations of the ten docking complexes with the lowest scores were visualized using PyMOL software.

Animal model and sample collection

All experiments were approved by the Institutional Animal Care and Use Committee of Sun Yat-Sen University (approval number: SYSU-IACUC-2023-000830) and were conducted in accordance with the National Institutes of Health Guide for the Care and Use of Laboratory Animals. Low-density lipoprotein receptor null (*Ldlr*$^{-/-}$) mice with a C57BL/6J background were purchased from GemPharmatech company (Jiangsu, China) and raised in the Laboratory Animal Center of Sun Yat-sen University (Guangzhou, China) until further experiments. Six-week-old *Ldlr*$^{-/-}$ mice were randomly (simple randomization) assigned to two groups. Only male mice were used in this study to avoid estrogen-induced variability in the atherosclerosis extent[16, 17]. The investigators were unaware of the allocation. Adeno-associated virus 9 (AAV9) carrying full-length human HDRACA (AAV-HDRACA) and negative control AAV9 (AAV-NC) were designed and purchased from HANBIO company. AAV9 vectors were modified by incorporating an endothelial cell-specific TIE1 promoter sequence to enhance the specificity of target gene expression in endothelial cells (ECs). AAV-NC and AAV-HDRACA were intravenously injected into *Ldlr*$^{-/-}$ mice. These groups were continuously fed a high-fat diet continuously for 12 weeks. Finally, mice were euthanized for further analysis.

FISH and immunofluorescence staining assays in aortic root sections

FISH assays were performed to detect HDRACA expression in ECs of aortic root sections using the Ribo™ Fluorescent In Situ Hybridization Kit according to the manufacturer's instructions. Briefly, samples were fixed with RNAase-free 4% paraformaldehyde for 15 min, embedded in Tissue-Tek optimal cutting temperature (OCT) compound (Cat. 4583, Japan, Sakura), and cut into 10-μm sections using a cryostat (NX50, Thermo Fisher Scientific, USA). After permeabilization with 0.5% TritonX-100, the samples were hybridized with Cy3-labeled HDRACA detection probes (customized by RiboBio) at 53 °C overnight. Then, the samples were subsequently subjected to immunofluorescence staining for CD31 as previously described with modifications [1, 18]. After being blocked with 5% BSA and 5% normal goat serum in

PBS for 1 h, the sections were incubated with primary antibodies against CD31 (Cat. ab7388, UK, Abcam) at 4 °C overnight, followed by 1 h incubation with Alexa Fluor 488-conjugated secondary antibody (Cat. ab150157, UK, Abcam) at room temperature. Finally, nuclei were counterstained with DAPI, and images were captured using a confocal laser scanning microscope equipped with a core data acquisition system (Nikon Eclipse Ni-E, Japan).

Immunofluorescence staining in whole mount en face mouse aortic preparations

Immunofluorescence staining of whole-mount en face mouse aortic preparations was performed as previously described[19]. Mice were anesthetized prior to thoracotomy, followed by intracardiac perfusion with 50 mM KCl and 4% paraformaldehyde. The aortas were then excised, defatted, longitudinally dissected, and pinned flat on silicone-coated plates. After being fixed in 4% paraformaldehyde at 4 °C overnight, aortas were permeabilized and blocked with PBS containing 5% BSA, 5% normal goat serum, and 0.3% Triton X-100 for 1 h. 1-2 thoracic aortic segments per animal were processed using floating immunostaining with primary antibodies against HSPB1 (1:200, Cat. MA5-32473, USA, Invitrogen), VCAM1 (1:250, Cat. ab134047, UK, Abcam), ICAM1 (1:50, Cat. ab222736, UK, Abcam), MCP-1 (1:100, Cat. ab315478, UK, Abcam) or vascular endothelial cadherin (VE-cadherin, 1:400, Cat. 14-1441-82, USA, Invitrogen), followed by incubation for 1 h with Alexa Fluor 647-conjugated secondary antibody (Cat. ab150075, UK, Abcam) or Alexa Fluor 488-conjugated secondary antibody (Cat. ab150157, UK, Abcam) at room temperature. Finally, the nuclei were counterstained with DAPI, and the specimens were mounted on a microscope slide. Images were captured using a confocal laser scanning microscope equipped with a core data acquisition system (LSM800, Zeiss, Germany).

Aortic Oil Red O Staining

Oil Red O staining in the aortic roots and en face was performed to evaluate atherosclerotic content as previously described with modifications [20]. Oil Red O (0.5 g) was dissolved in 1000 ml of isopropanol by heating until it was fully dissolved. After cooling to room temperature, the solution was filtered through filter paper to obtain an Oil Red O storage solution. The storage solution was mixed with double-distilled water at a 3:2 ratio and filtered through filter paper to obtain the Oil Red O working solution.

For Oil Red O staining of aortic root sections, 5-μm thick cryosections of the aortic root were

immersed in freshly prepared Oil Red O working solution for 1 h. Then, the sections were washed off excess dye with 60% isopropanol, stained with hematoxylin for approximately 30 s, and rinsed three times with double-distilled water. Finally, the slides were examined using a pathological section scanner (KF-PRO-020, Kfbio, China).

For Oil Red O staining in whole-mount en face mouse aortic preparations, the entire aortas were isolated from the ascending aortas to the bilateral common iliac arteries, including the three branches of the aortic arches. Aortas were fixed in 10% formalin for 24 h. After the adventitial tissues were removed, the aortas were opened longitudinally and stained with freshly prepared Oil Red O working solution for 1 h. Then, the aortas were washed off the excess stain with 60% isopropanol and rinsed three times with double-distilled water. After fixation on a black rubber surface, the aortas were observed and photographed under a stereomicroscope (M205FA, Leica, Germany).

Immunohistochemistry

Immunohistochemistry assays were performed as previously described with modifications [1]. OCT-embedded sections were thawed at room temperature and hydrated. After being heated in pH 9.0, ethylenediaminetetraacetic acid antigen retrieval solution (Cat. G1203, China, Servicebio) to retrieve antigens, the sections were incubated in a 3% hydrogen peroxide solution at room temperature, protected from light, for 25 min to block endogenous peroxidase activity. Then, the sections were blocked with 5% normal goat serum for 30 min and further incubated overnight at 4 °C with the F4/80 antibody (1:5000, Cat. 28463-1-AP, USA, Proteintech). This was followed by a 50-min incubation with an HRP-labeled secondary antibody (Cat. GB23303, China, Servicebio). Color was developed using diaminobenzidine. The sections were counterstained with hematoxylin, dehydrated in ethanol, and cleared with xylene. Finally, images were captured using a pathological section scanner (KF-PRO-020, Kfbio, China).

Statistical analysis

Data are expressed as mean ± standard deviation (SD). Statistical analyses were conducted using SPSS version 13.0 or GraphPad Prism 9.0. Data were first tested for normality. For normally distributed data, differences among groups were assessed using unpaired two-tailed Student's t-test for comparisons between two groups and one-way or two-way ANOVA

followed by Tukey's multiple comparisons test for comparisons among multiple groups. For data that did not conform to a normal distribution, the Mann-Whitney *U* test was used for two groups, and the Kruskal-Wallis test followed by Dunn's multiple comparisons test was used for multiple groups. The chi-square test was used to compare age and smoking status between CAD patients and healthy controls. Statistical significance was set at $p<0.05$.

Reference

1. Mo ZW, Peng YM, Zhang YX, Li Y, Kang BA, Chen YT, et al. High-density lipoprotein regulates angiogenesis by long non-coding RNA HDRACA. Signal transduction and targeted therapy. 2023; 8: 299.

2. Liu X, Wang TT, Li Y, Shi MM, Li HM, Yuan HX, et al. High density lipoprotein from coronary artery disease patients caused abnormal expression of long non-coding RNAs in vascular endothelial cells. Biochemical and biophysical research communications. 2017; 487: 552-9.

3. Li HM, Mo ZW, Peng YM, Li Y, Dai WP, Yuan HY, et al. Angiogenic and Antiangiogenic mechanisms of high density lipoprotein from healthy subjects and coronary artery diseases patients. Redox biology. 2020; 36: 101642.

4. Kang BA, Li HM, Chen YT, Deng MJ, Li Y, Peng YM, et al. High-density lipoprotein regulates angiogenesis by affecting autophagy via miRNA-181a-5p. Science China Life sciences. 2024; 67: 286-300.

5. Sasko B, Scharow L, Mueller R, Jaensch M, Dammermann W, Seibert FS, et al. Reduced high-density lipoprotein antioxidant function in patients with coronary artery disease and acute coronary syndrome. JCI insight. 2025; 10.

6. Chen X, Zaro JL, Shen WC. Fusion protein linkers: property, design and functionality. Advanced drug delivery reviews. 2013; 65: 1357-69.

7. Yuan HX, Chen YT, Li YQ, Wang YS, Ou ZJ, Li Y, et al. Endothelial extracellular vesicles induce acute lung injury via follistatin-like protein 1. Science China Life sciences. 2024; 67: 475-87.

8. Liu ZL, Li Y, Lin YJ, Shi MM, Fu MX, Li ZQ, et al. Aging aggravates aortic aneurysm and dissection via miR-1204-MYLK signaling axis in mice. Nature communications. 2024; 15: 5985.

9. Chen S, Gao JJ, Liu YJ, Mo ZW, Wu FY, Hu ZJ, et al. The oxidized phospholipid PGPC impairs endothelial function by promoting endothelial cell ferroptosis via FABP3. Journal of lipid research. 2024; 65: 100499.

10. Gong K, Song K, Wang H, Li L, Sun X, Sun L, et al. Triptonide-mediated PTGS2 Inhibition Induces Autophagic Cell Death to Suppress the Progression of Triple-negative Breast

Cancer and Epithelial Ovarian Cancer. International journal of biological sciences. 2026; 22: 2215-32.

11. Li Y, Chen YT, Liu JS, Liang KF, Song YK, Cao Y, et al. Oncoprotein-induced transcript 3 protein-enriched extracellular vesicles promotes NLRP3 ubiquitination to alleviate acute lung injury after cardiac surgery. Journal of molecular and cellular cardiology. 2024; 195: 55-67.

12. Gao JJ, Wu FY, Liu YJ, Li L, Lin YJ, Kang YT, et al. Increase of PCSK9 expression in diabetes promotes VEGFR2 ubiquitination to inhibit endothelial function and skin wound healing. Science China Life sciences. 2024; 67: 2635-49.

13. Xiao R, Lu X, Huang F, Zhao Y, Jin H, Jia X, et al. METTL3-mediated m6A methylation on lncRNA H19 inhibits intrahepatic cholangiocarcinoma progression through PPARγ downregulation. International journal of biological sciences. 2025; 21: 6062-80.

14. Lu Q, Lv X, Wang J, Xia B, Yan H, Wang Z, et al. Methylation-mediated LncRNA CRAT40 promotes colorectal cancer progression by recruiting YBX1 to initiate RelA transcription. International journal of biological sciences. 2025; 21: 4834-50.

15. Li RH, Tian T, Ge QW, He XY, Shi CY, Li JH, et al. A phosphatidic acid-binding lncRNA SNHG9 facilitates LATS1 liquid-liquid phase separation to promote oncogenic YAP signaling. Cell research. 2021; 31: 1088-105.

16. Dai X, Liu S, Cheng L, Huang T, Guo H, Wang D, et al. Epigenetic Upregulation of H19 and AMPK Inhibition Concurrently Contribute to S-Adenosylhomocysteine Hydrolase Deficiency-Promoted Atherosclerotic Calcification. Circulation research. 2022; 130: 1565-82.

17. Zheng L, Chen X, He X, Wei H, Li X, Tan Y, et al. METTL4-Mediated Mitochondrial DNA N6-Methyldeoxyadenosine Promoting Macrophage Inflammation and Atherosclerosis. Circulation. 2025; 151: 946-65.

18. Liu Z, Cao Y, Liao XL, Ou ZJ, Mo ZW, Liu YF, et al. Apolipoprotein A-I Mimetic Peptide Restores VEGF-induced Angiogenesis in Hypercholesterolemic Ischemic Heart by Reducing HDL Proinflammatory Properties. J Cardiovasc Transl Res. 2025; 18: 58-69.

19. Hamczyk MR, Nevado RM, Gonzalo P, Andrés-Manzano MJ, Nogales P, Quesada V, et al. Endothelial-to-Mesenchymal Transition Contributes to Accelerated Atherosclerosis in Hutchinson-Gilford Progeria Syndrome. Circulation. 2024; 150: 1612-30.

20. Ning DS, Ma J, Peng YM, Li Y, Chen YT, Li SX, et al. Apolipoprotein A-I mimetic peptide inhibits atherosclerosis by increasing tetrahydrobiopterin via regulation of GTP-cyclohydrolase 1 and reducing uncoupled endothelial nitric oxide synthase activity. Atherosclerosis. 2021; 328: 83-91.

Supplementary Table S1. Characteristics of subjects for isolating HDL

	CAD (n=50)	Healthy Controls (n=50)	p-value
Male[no.(%)]	27 (54.00)	25 (50.00)	0.69
Age[years]	54.64 ± 5.56	54.42 ± 8.65	0.88
Smoking[no.(%)]	17 (34.00)	11 (22.00)	0.18
Body mass index[kg/m^2]	25.14 ± 3.12	23.39 ± 3.43	<0.05
HbA1c[%]	5.55 ± 0.23	5.48 ± 0.25	0.14
Heart rate[bpm]	70.44 ± 11.53	72.82 ± 10.44	0.21
Mean arterial pressure[mmHg]	89.68 ± 9.03	88.50 ± 9.92	0.54
Total cholesterol[mmol/L]	4.81 ± 1.10	4.34 ± 0.62	<0.05
Triglycerides[mmol/L]	1.79 ± 0.74	1.09 ± 0.32	<0.05
LDL cholesterol[mmol/L]	3.09 ± 0.87	2.67 ± 0.44	<0.05
HDL cholesterol[mmol/L]	1.08 ± 0.21	1.22 ± 0.26	<0.05
HDL bound S1P[pmol/mg]	94.69 ± 38.18	295.51 ± 60.19	<0.05
Creatinine[mmol/L]	72.46 ± 16.29	70.16 ± 14.03	0.45

CAD: coronary artery disease; HbA1c: Hemoglobin A1c; HDL: high-density lipoprotein; LDL: low-density lipoprotein; S1P: sphingosine-1-phosphate.

Supplementary Table S2. Target sequences for lncRNA Smart Silencers and siRNAs

HDRACA lncRNA Smart Silencer	ASO-1	AAGGCTGGTGTCTGATTGAT
	ASO-2	GCTGTCTAAGCTAATGTGGA
	ASO-3	CCCAAAGCAGGAGCGTCTCA
	siRNA-1	GGGAACCTATTCCTAGATA
	siRNA-2	CCAACCTTTAGATTCAGCT
	siRNA-3	GAACCTTCTTTGGTACAAA
HSPB1 siRNAs	siRNA-1	GCTGCAAAATCCGATGAGA
	siRNA-2	GGTGCTTCACGCGGAAATA
	siRNA-3	TGACGGTCAAGACCAAGGA

Supplementary Table S3. Primers sequences for lncRNA and mRNA RT-qPCR

Target	**Forward**	**Reverse**
HDRACA	GTGGCTTAGGCCAGGTACAAC	GTGTCGATGGATCGGGGTC
ACTB	TCAAGATCATTGCTCCTCCTGAG	ACATCTGCTGGAAGGTGGACA
GAPDH	CAGGAGGCATTGCTGATGAT	GAAGGCTGGGGCTCATTT

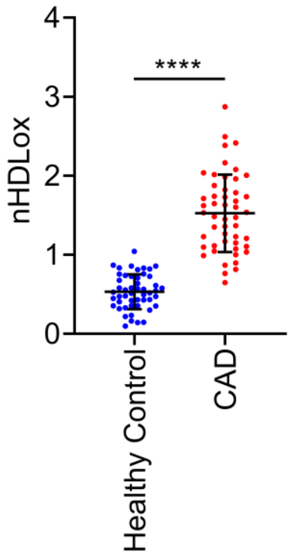

Supplementary Figure S1. nHDLox levels are significantly higher in patients with CAD than in healthy controls

Quantification of nHDLox levels in healthy controls (n=50) and patients with CAD (n=50). Data are presented as mean ± SD. Statistical significance was determined using unpaired Student's t-test. ****$p<0.0001$.

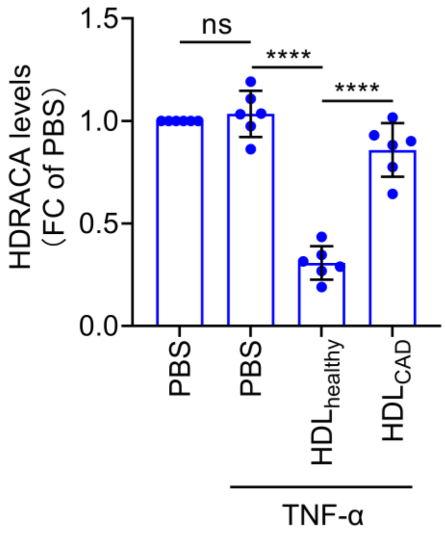

Supplementary Figure S2. HDL$_{healthy}$ significantly downregulates HDRACA levels in TNF-α treated-HUVECs, whereas HDL$_{CAD}$ is less effective

RT-qPCR confirmed the expression of HDRACA in HUVECs treated with PBS, HDL$_{healthy}$ or HDL$_{CAD}$ for 24 h, followed by 6 h incubation with TNF-α. Data are presented as mean ± SD. n=6. Statistical significance was determined using one-way ANOVA with Tukey's multiple comparisons test. ****p<0.0001; ns, not significant.

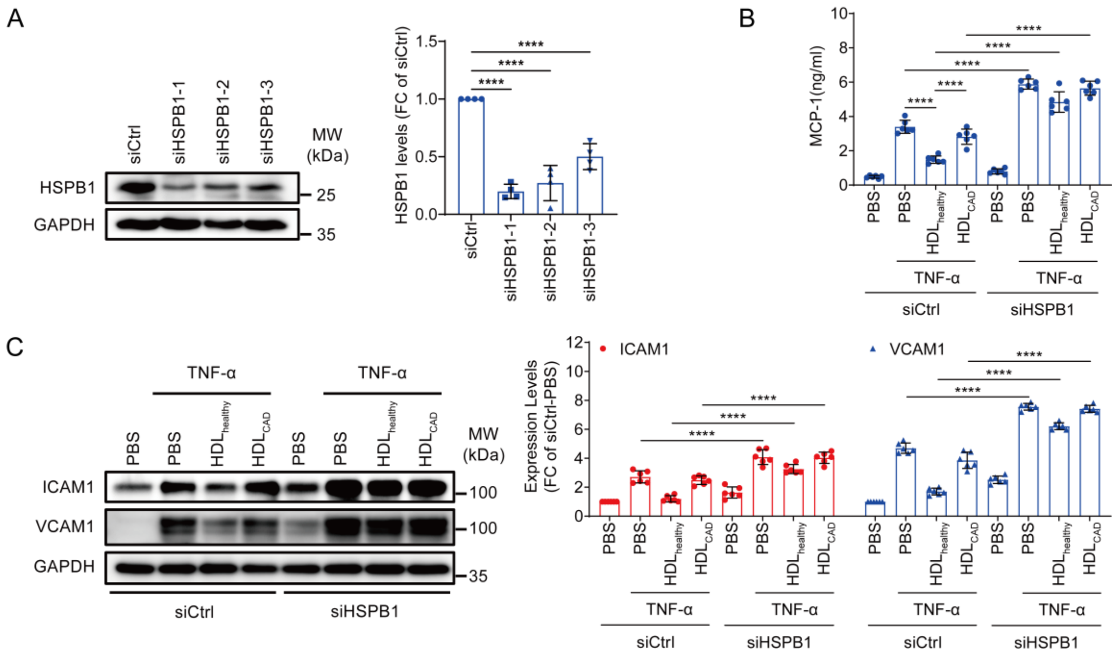

Supplementary Figure S3. HSPB1 knockdown inhibits adhesive and chemotactic effects of HUVECs

A. Western blot analysis of the expression of HSPB1 in HUVECs transfected with negative control siRNA or HSPB1 siRNAs. Representative blots (left) and quantifications (right) are shown. B. Quantification of ELISA analysis of MCP-1 from HUVECs treated with $HDL_{healthy}$ or HDL_{CAD} for 24 h followed by 6 h incubation with TNF-α after HSPB1 knockdown. C. Representative blots (left) and quantification (right) of western blot analysis of ICAM1 and VCAM1 in HUVECs treated with $HDL_{healthy}$ or HDL_{CAD} for 24 h, followed by 6 h incubation with TNF-α after HSPB1 knockdown. Data are presented as mean ± SD. For A, n=4; For B and C, n=6. Statistical significance was determined using one-way ANOVA with Tukey's multiple comparisons test (A) and two-way ANOVA with Tukey's multiple comparisons test (B, C). ****$p<0.0001$.

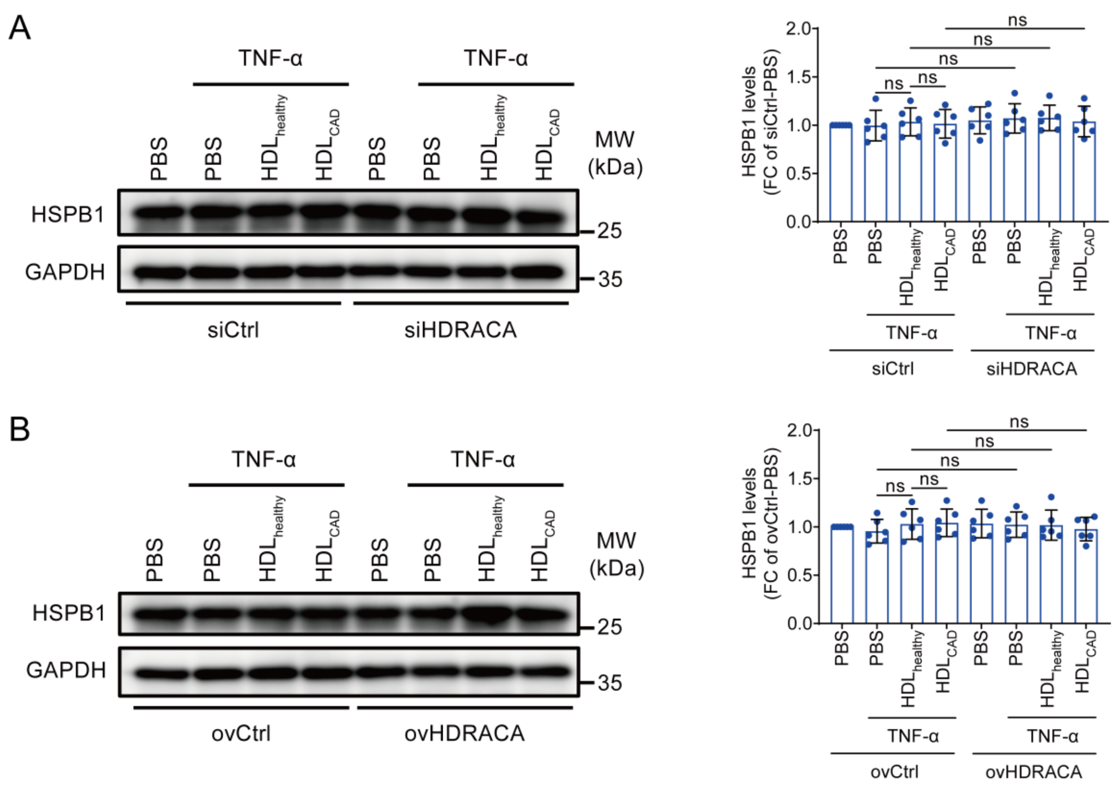

Supplementary Figure S4. Neither HDLhealthy and HDLCAD treatment nor silencing and overexpression of HDRACA affect HSPB1 levels in HUVECs

A. Western blot analysis of HSPB1 levels in HUVECs treated with HDLhealthy or HDLCAD followed by incubation with TNF-α after silencing of HDRACA. Representative blots (left) and quantification (right) are shown. B. Western blot analysis of HSPB1 levels in HUVECs treated with HDLhealthy or HDLCAD followed by incubation with TNF-α after overexpression of HDRACA. Representative blots (left) and quantification (right) are shown. Data are presented as mean ± SD. n=6. Statistical significance was determined using two-way ANOVA with Tukey's multiple comparisons test (A, B). ns, not significant.

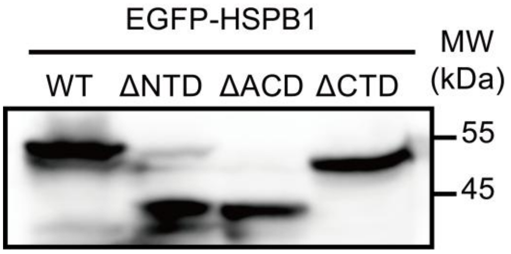

Supplementary Figure S5. EGFP-linked HSPB1-WT and EGFP-linked HSPB1 truncated mutants express successfully in HUVECs

Western blot analysis of EGFP-linked HSPB1 wild-type (WT), EGFP-linked HSPB1 lacking N-terminal domain (ΔNTD), EGFP-linked HSPB1 lacking α-crystallin domain (ΔACD), and EGFP-linked HSPB1 lacking C-terminal domain (ΔCTD) in HUVECs transduced with lentivirus for 72 h. n=4.

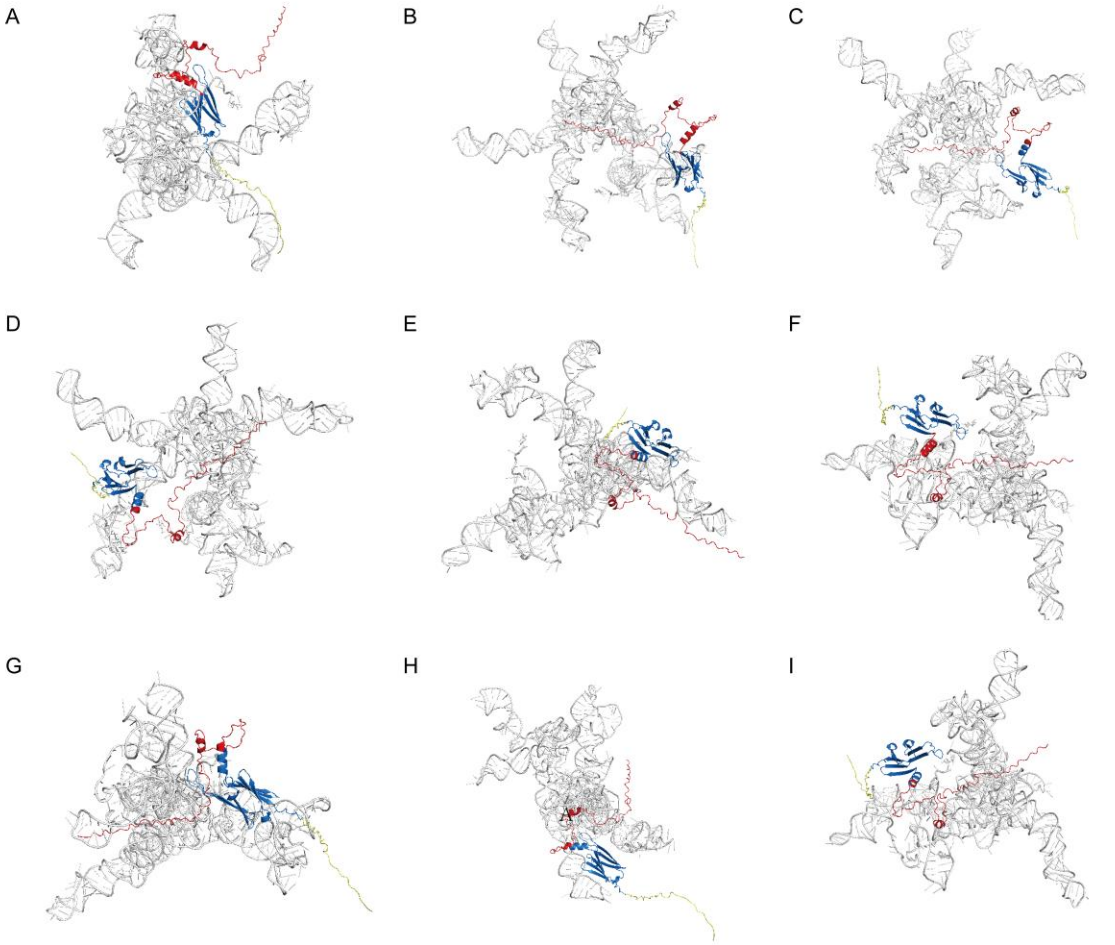

Supplementary Figure S6. Molecular docking results of interaction between HDRACA and HSPB1

The conformations of the nine docking complexes with the second to ninth lowest scores are shown from A to I.

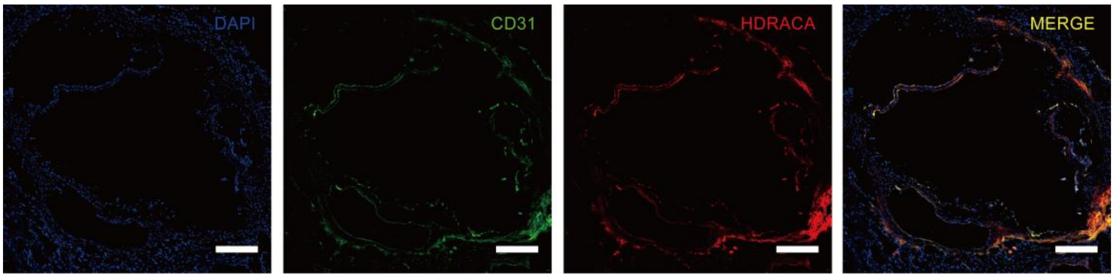

Supplementary Figure S7. HDRACA was successfully expressed in mouse aortic endothelial cells

FISH-immunofluorescence showed colocalization of HDRACA (red) and CD31 (green) in the aortic root of *Ldlr*$^{-/-}$ mice fed a high-fat diet for 12 weeks after transduction with AAV-HDRACA. The nuclei were stained with DAPI (blue). Scale bars, 200 µm.
